# Resetting Transcription Factor Control Circuitry toward Ground-State Pluripotency in Human

**DOI:** 10.1016/j.cell.2015.06.052

**Published:** 2015-07-16

**Authors:** Yasuhiro Takashima, Ge Guo, Remco Loos, Jennifer Nichols, Gabriella Ficz, Felix Krueger, David Oxley, Fatima Santos, James Clarke, William Mansfield, Wolf Reik, Paul Bertone, Austin Smith

(Cell *158*, 1254–1269; September 11, 2014)

Our paper reported that short-term expression of NANOG and KLF2 is sufficient to reset the state of pluripotency in human embryonic stem cells. Figure 7A, depicting colony formation after induced transgene expression, contains two identical images in which the same well is shown to represent both H9 and H1 cells (top row, images 4 and 5). This duplication arose during consolidation of images into the composite figure where the H9 well image was inadvertently inserted for both panels. The revised Figure 7 containing the correct H1 well image is shown below, and the figure has been corrected online.

**Figure 7:**
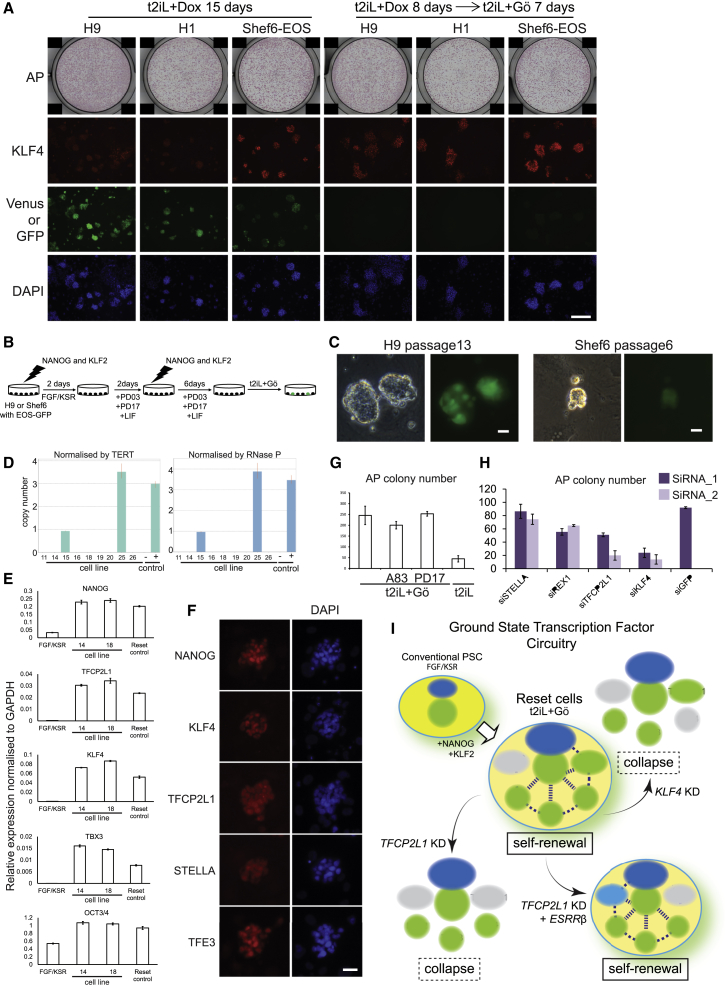
Resetting by Transient Transgenesis

