# Supplementary material for: Resetting Transcription Factor Control Circuitry toward Ground-State Pluripotency in Human
Source: Cell. 2014 Sep 11;158(6):1254–69. doi: 10.1016/j.cell.2014.08.029 (PMC4162745; doi:10.1016/j.cell.2014.08.029)
Supplement: Document S1. Article plus Supplemental Information [file mmc5.pdf]

Cell

Resetting Transcription Factor Control Circuitry toward Ground-State Pluripotency in Human

Yasuhiro Takashima,[1,2] Ge Guo,[1] Remco Loos,[1,3] Jennifer Nichols,[1,4] Gabriella Ficz,[5] Felix Krueger,[6] David Oxley,[6] Fatima Santos,[6] James Clarke,[1] William Mansfield,[1] Wolf Reik,[6,7,8] Paul Bertone,[1,3,9,*] and Austin Smith[1,10,*]
[1]Wellcome Trust–Medical Research Council Stem Cell Institute, University of Cambridge, Tennis Court Road, Cambridge CB2 1QR, UK
[2]PRESTO, Japan Science and Technology Agency, 4-1-8 Honcho, Kawaguchi, Saitama, 332-0012, Japan
[3]European Molecular Biology Laboratory, European Bioinformatics Institute, Wellcome Trust Genome Campus, Hinxton CB10 1SD, UK
[4]Department of Physiology, Development, and Neuroscience, University of Cambridge, Tennis Court Road, Cambridge CB2 3EG, UK
[5]Centre for Haemato-Oncology, Barts Cancer Institute, University of London, Charterhouse Square, London EC1M 6BQ, UK
[6]Babraham Institute, Babraham, CB22 3AT, UK
[7]Centre for Trophoblast Research, University of Cambridge, Tennis Court Road, Cambridge CB2 3EG, UK
[8]Wellcome Trust Sanger Institute, Wellcome Trust Genome Campus, Hinxton CB10 1SA, UK
[9]Genome Biology and Developmental Biology Units, European Molecular Biology Laboratory, Meyerhofstraβe 1, 69117 Heidelberg, Germany
[10]Department of Biochemistry, University of Cambridge, Tennis Court Road, Cambridge CB2 1GA, UK
*Correspondence: bertone@ebi.ac.uk (P.B.), austin.smith@cscr.cam.ac.uk (A.S.)
http://dx.doi.org/10.1016/j.cell.2014.08.029
This is an open access article under the CC BY license (http://creativecommons.org/licenses/by/3.0/).

SUMMARY

Current human pluripotent stem cells lack the transcription factor circuitry that governs the ground state of mouse embryonic stem cells (ESC). Here, we report that short-term expression of two components, NANOG and KLF2, is sufficient to ignite other elements of the network and reset the human pluripotent state. Inhibition of ERK and protein kinase C sustains a transgene-independent rewired state. Reset cells self-renew continuously without ERK signaling, are phenotypically stable, and are karyotypically intact. They differentiate in vitro and form teratomas in vivo. Metabolism is reprogrammed with activation of mitochondrial respiration as in ESC. DNA methylation is dramatically reduced and transcriptome state is globally realigned across multiple cell lines. Depletion of ground-state transcription factors, *TFCP2L1* or *KLF4*, has marginal impact on conventional human pluripotent stem cells but collapses the reset state. These findings demonstrate feasibility of installing and propagating functional control circuitry for ground-state pluripotency in human cells.

INTRODUCTION

Human pluripotent stem cells (PSC), derived from supernumerary embryos or by molecular reprogramming, show several distinguishing characteristics compared with paradigmatic mouse embryonic stem cells (ESC). Originally regarded as inconsequential species-specific features, increasing evidence suggests discrete developmental identities. Notably, derivation of postimplantation epiblast stem cells (EpiSC) (Brons et al., 2007; Tesar et al., 2007) shows that alternative pluripotent stem cells can be obtained from mice. EpiSC are related to primitive streak-stage late epiblast (Kojima et al., 2014; Tsakiridis et al., 2014). The terminology naive and primed was introduced to describe early and late phases of epiblast ontogeny and respective ESC and EpiSC derivatives (Nichols and Smith, 2009). Human PSC are considered more related to primed EpiSCs than to naive ESC.

Mouse ESC self-renewal is favored by blockade of mitogen-activated protein kinase (Erk) signaling and is stimulated by the cytokine leukemia inhibitory factor (LIF) (Nichols and Smith, 2012). Combining two inhibitors (2i) of the Erk pathway and of glycogen synthase kinase-3 with LIF (2iL) provides a defined culture system that is effective for all strains of mouse and rat tested, supporting efficient ESC derivation and clonal expansion from dissociated cells (Boroviak et al., 2014; Ying et al., 2008). This serum- and growth-factor-free formulation is also selective; most cell types, including EpiSC and human PSC, differentiate or die in 2iL alone. The stability and relative homogeneity of ESC in 2iL (Wray et al., 2010) is postulated to represent a developmental ground state closely reflective of the newly formed epiblast in the mature blastocyst (Nichols and Smith, 2009). In contrast, EpiSC and human PSC are heterogeneous between and within cell lines (Kojima et al., 2014; Tsakiridis et al., 2014) and passage poorly when dissociated, resulting in low cloning efficiency. They are unresponsive to LIF but rely on growth factors, specifically fibroblast growth factor (FGF) and TGFβ/activin (Amit et al., 2000; Vallier et al., 2005).

The transcriptional regulators Oct4 and Sox2 constitute the pillar of pluripotency through all its phases. These factors are

essential but are not restricted to, nor sufficient for, the ESC ground state. A select group of regulators present in the preimplantation epiblast and ESC interconnect with Oct4/Sox2 to confer and sustain naive status. Foremost among these are Nanog, Klf2, Klf4, Esrrb, and Tfcp2l1 (Dunn et al., 2014; Ivanova et al., 2006; Martello et al., 2013; Niwa et al., 2009; Ye et al., 2013). Apart from Nanog, these factors are expressed at very low levels or are absent from EpiSC and human PSC. Strikingly, however, transfection of EpiSC with a single component in conjunction with transfer to 2iL can ignite the entire circuitry and reset the ESC ground state (Guo et al., 2009; Hanna et al., 2009; Silva et al., 2009).

Conversion of mouse EpiSCs to ESC may provide a paradigm for generation of human ground-state PSC. Early trials (Hanna et al., 2010; Wang et al., 2011) noted ESC-like morphology, but cells appeared unstable. More recently, complex culture formulations have been proposed to allow propagation of human PSC with altered characteristics (Chan et al., 2013; Gafni et al., 2013; Ware et al., 2014), but these cells remain dependent on FGF, TGFβ, and/or serum replacement factors and lack evidence for rewiring of transcriptional control circuitry. We therefore investigated further the generation and stabilization of human cells with phenotypic features and transcription factor governance characteristic of ground-state pluripotency.

RESULTS

NANOG and KLF2 Reset the Human PSC Phenotype

Expression of *Nanog* or *Klf2* converts mouse EpiSC to ground-state ESC in 2iL (Hall et al., 2009; Silva et al., 2009). We tested the effect of this pair of factors in human embryo-derived H9 cells. We introduced doxycycline (DOX)-inducible *KLF2* and *NANOG*/Venus constructs along with an rtTA vector. Transfectants were selected in conventional PSC culture medium (FGF/KSR) without DOX. Cultures were replated prior to addition of DOX. DOX-induced cells differentiated or died in FGF/KSR. In contrast, in 2iL undifferentiated cells persisted and formed colonies that could readily be expanded. These cells were positive for Venus, indicating robust transgene induction, and displayed the tightly packed domed appearance typical of mouse ESC in 2iL (Figure 1A). Cultures could be propagated continuously by enzymatic dissociation to single cells without requirement for ROCKi (Watanabe et al., 2007). On withdrawal of DOX, however, cultures degenerated unless transferred into FGF/KSR when they reverted to conventional flat PSC colony morphology and sensitivity to dissociation. Cells could be cycled between these two exclusive conditions (Figure 1A).

We investigated candidate pathways for the ability to support propagation in 2iL upon DOX withdrawal. Addition of the protein kinase C (PKC) inhibitor Gö6983 (5 μM), which suppresses mouse ES cell differentiation (Dutta et al., 2011), sustained compact refractile colonies lacking Venus (Figure 1B). These cultures expressed OCT4 (Figure S1 available online) and expanded continuously, although proliferation was reduced and morphology less consistent compared to cells in DOX. Moderating GSK3 inhibition improves rat ES cell culture (Chen et al., 2013; Meek et al., 2013), and we also observed that combination of Gö6983 with GSK3 inhibitor CH is unfavorable for mouse ESC propagation (A.S., unpublished data). Colony morphology in the absence of DOX was improved without CH, but growth rate was reduced. An intermediate concentration of 1 μM CH restored growth while maintaining morphology (Figure 1C). Henceforth cells were maintained in *t*itrated 2i with LIF and Gö6983 (t2iL+Gö). Undifferentiated colonies formed from dissociated cells without ROCKi (Figure 1D). Immunoblotting confirmed that Erk signaling was fully blocked (Figure S1B). Colony formation was not suppressed by inhibitors of TGF-β/activin or FGF receptors (Figures 1E and S1C).

We induced conversion using different embryo-derived and induced PSC (Table S1) and in all cases obtained abundant tightly packed colonies with DOX. On DOX withdrawal and switch to t2iL+Gö, cultures initially became heterogeneous. Tightly packed colonies dominated after two to four passages and thereafter were readily maintained over multiple passages by single-cell dissociation every 4–6 days and replating at a split ratio of 1:3 to 1:5. Independent cultures were propagated for more than 20 passages (4 months) with no deterioration in morphology or doubling time (Figure 1F and Table S1). Metaphase counts and array analyses (Figure 1G and Table S1) confirmed genetic integrity of different lines over multiple passages.

Following DOX withdrawal, transgene products were undetectable by fluorescence or qRT-PCR (Figures S1D and S1E). We profiled cultures in t2iL+Gö for the suite of transcription factors diagnostic of, and functionally implicated in, the ESC ground state (Dunn et al., 2014). Compared with no or minimal expression in conventional PSC, all factors were substantially upregulated apart from ESRRB (Figures 1H and S1E*).* Protein expression was confirmed by immunostaining and immunoblotting (Figures 1I, 1J, and S1F). TFE3 was nuclear compared with cytoplasmic localization in conventional PSC, as shown for mouse naive versus primed cells (Betschinger et al., 2013).

The preceding experiments were performed on feeders. From observations with mouse ESC, we realized that, without buffering by feeders, Gö was potentially toxic. We therefore reduced Gö to 2 μM and also added ROCKi prior to passaging. In these conditions, reset cells could form undifferentiated colonies on Matrigel or laminin 511-E8 (Nakagawa et al., 2014) (Figure 1K). On both substrates, cells could be expanded, albeit more slowly on laminin, with retained expression of ground-state pluripotency factors (Figure 1L).

These observations indicate that NANOG and KLF2 can reset self-renewal requirements and transcription factor complement in human PSC and, furthermore, that this rewired state may be rendered independent of transgene expression by fine-tuning 2iL in combination with the PKC inhibitor Gö6983.

Differentiation Competence

To test whether reset PSC are capable of germ-layer specification, we generated embryoid bodies directly from reset cells. Embryoid bodies were harvested after 5 or 10 days and analyzed by qRT-PCR. Transcripts diagnostic of the three germ layers were upregulated (Figure 2A). Differentiation capacity was tested further by grafting to NOD/SCID mice. Cells transplanted directly from t2iL+Gö formed teratomas by 12 weeks that contained

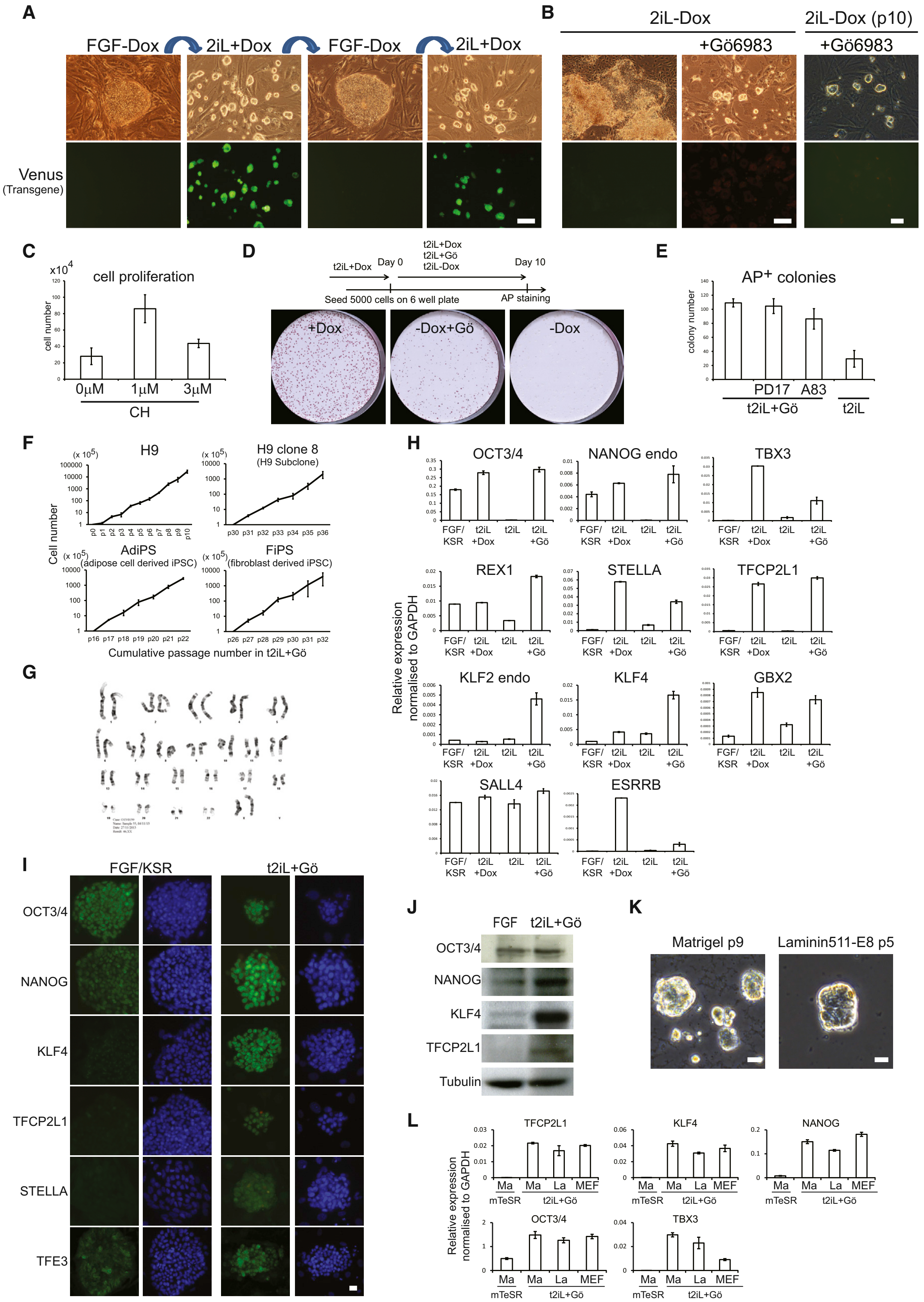

(legend on next page)

well-differentiated regions of neuroepithelium, cartilage, and digestive tract (Figure 2B).

On transfer to FGF/KSR, reset cells adopted a conventional PSC phenotype (Figure 2C) accompanied by downregulation of ground-state pluripotency factors (Figure 2D). After culture for two passages in FGF/KSR, cells lost ability to form colonies in t2iL+Gö, confirming a stable change in cell state (Figure 2E). We reasoned that reset cells could be channeled into adherent differentiation by exposure to FGF/KSR and application of protocols developed for conventional PSC. Cells exchanged into FGF/KSR for a few days responded to activin and Wnt3A (Kroon et al., 2008) by differentiation into definitive endoderm (Figure 2F). Conversely, treatment with Noggin and SB431542 (Chambers et al., 2009) resulted in neuronal cells with dendritic processes (Figure 2G). Cells changed into FGF/KSR and aggregated, upregulated cardiac markers (Figure 2H), and formed outgrowths with beating foci.

We conclude that reset cells can progress via a primed state into germ-layer differentiation.

Mitochondrial and Metabolic Adjustment

ESC utilize oxidative phosphorylation, whereas EpiSC/human PSC are almost entirely glycolytic with very low mitochondrial respiration capacity (Zhou et al., 2012). We measured basal oxygen consumption rate (OCR) and found that it was substantially higher in reset cells than in conventional PSC (Figure 3A). Higher electron transport chain activity in reset cells was evidenced by a greater OCR increase in response to the mitochondrial uncoupler FCCP (Figure 3A). Reset cells also displayed intense staining with tetramethylrhodamine methyl ester (TMRE), indicative of mitochondrial membrane depolarization (Figure 3B). Furthermore, the complex IV cytochrome *c* oxidase (COX) gene family displayed higher expression in reset cells than conventional PSC for 14 out of 17 genes (Figure S2A), similar to findings for ESC and EpiSC (Zhou et al., 2012).

We examined functional consequences of altered metabolic properties by culture in 2-deoxyglucose to inhibit glycolysis and in reduced concentrations of glucose to increase dependency on mitochondrial respiration. Unlike conventional PSC, reset cells formed undifferentiated colonies in the presence of 2-deoxyglucose (Figure 3C) or as low as 0.2 mM glucose (Figure S2B).

These data indicate that resetting human PSC is accompanied by a profound mitochondrial activation and metabolic realignment.

Epigenetic Reorganization

Global DNA hypomethylation is a feature of early embryo cells that is recapitulated in ESC cultured in 2i in contrast to hypermethylation in EpiSCs (Ficz et al., 2013; Habibi et al., 2013; Leitch et al., 2013). Immunofluorescence staining for 5-methylcytosine (5mC) was notably weaker in reset cells than conventional cultures (Figure 4A). Mass spectrometric quantification confirmed a major reduction in total 5mC and also in 5-hydroxymethylcytosine (Figure 4B). Bisulfite sequencing (BS-seq) at 8.8× genome coverage (Figure S3A) substantiated more than 50% loss of CpG methylation genome wide (Figure 4C), along with lower non-CpG methylation. Demethylation was substantial in most genomic contexts (Figure 4D). A representative genomic interval shows hypomethylation across the *SOX2* locus (Figure S3B). A minor subset of genes showed retained or even increased methylation.

The X chromosome in reset cells exhibited specific reduction in intermediate levels of CGI demethylation (Figure 4E). Intermediate levels are likely to reflect methylation of a proportion of X-linked CGIs in conventional PSC. Consistent with epigenetic erasure of the X chromosome, we observed that foci of histone 3 lysine 27 trimethylation (H3K27me3) were almost entirely lacking in reset XX cells (Figure 4F), although as previously described (Silva et al., 2008; Tomoda et al., 2012), this modification was already absent in many of the parental cells. Notably, however, upon transfer of reset cells to KSR/FGF culture conditions, foci of H3K27me3 appeared in the majority of cells within two passages. We also examined trimethylation of histone 3 lysine 9 (H3K9me3) associated with gene silencing. Reset cells exhibit much lower levels of this feature compared with conventional human PSC, recapitulating the difference observed between mouse ESC and EpiSC (Figures 4G, S3C, and S3D).

These data indicate that resetting the human PSC state is accompanied by profound epigenetic deconstruction. Local

Figure 1. Resetting Human PSC

Data in this and other figures are from H9 cells unless otherwise indicated, but similar results were obtained from H1 and Shef6 cells and various iPS cell lines (Table S1 and Figure S1)

(A) Induction or silencing of transgenes combined with switching between 2iL and FGF/KSR supports expansion of colonies with distinct morphology. Transgene expression indicated by the Venus reporter.

(B) PKC inhibitor Gö6983 maintains colony morphology in the absence of transgene expression. Intrinsic fluorescence of Gö produces a faint red background signal.

(C) Expansion in different CH concentrations. Cells were plated at 5×10^4 cells per well and were cultured for 4 days in PD03 with LIF and 0, 1, or 3 μM CH.

(D) Cells previously cultured in t2iL with DOX were plated in 6-wells without ROCKi in conditions indicated and were stained after 10 days.

(E) Cells maintained in t2iL+Gö were seeded in 12-well dishes without ROCKi in conditions indicated.

(F) Cell proliferation data.

(G) G-banded karyotype of reset cells at passage 16 (converted at parental passage 40).

(H) qRT-PCR for pluripotency factor transcripts.

(I) Immunostaining for ground-state pluripotency markers. The dot in the TFCP2L1 image of reset cells is due to intrinsic fluorescence of Gö.

(J) Immunoblotting for ground-state pluripotency proteins in conventional and reset cells.

(K) Reset cells on Matrigel or laminin 511-E8.

(L) qRT-PCR for ground-state transcription factor transcripts in reset cells after five passages on Matrigel (Ma) or Laminin511-E8 (La). Cultures on MEF and conventional PSC on Matrigel in mTeSR are controls.

Scale bars: (A and B) 100 μM, (I and K) 20 μM. Error bars indicate SD.

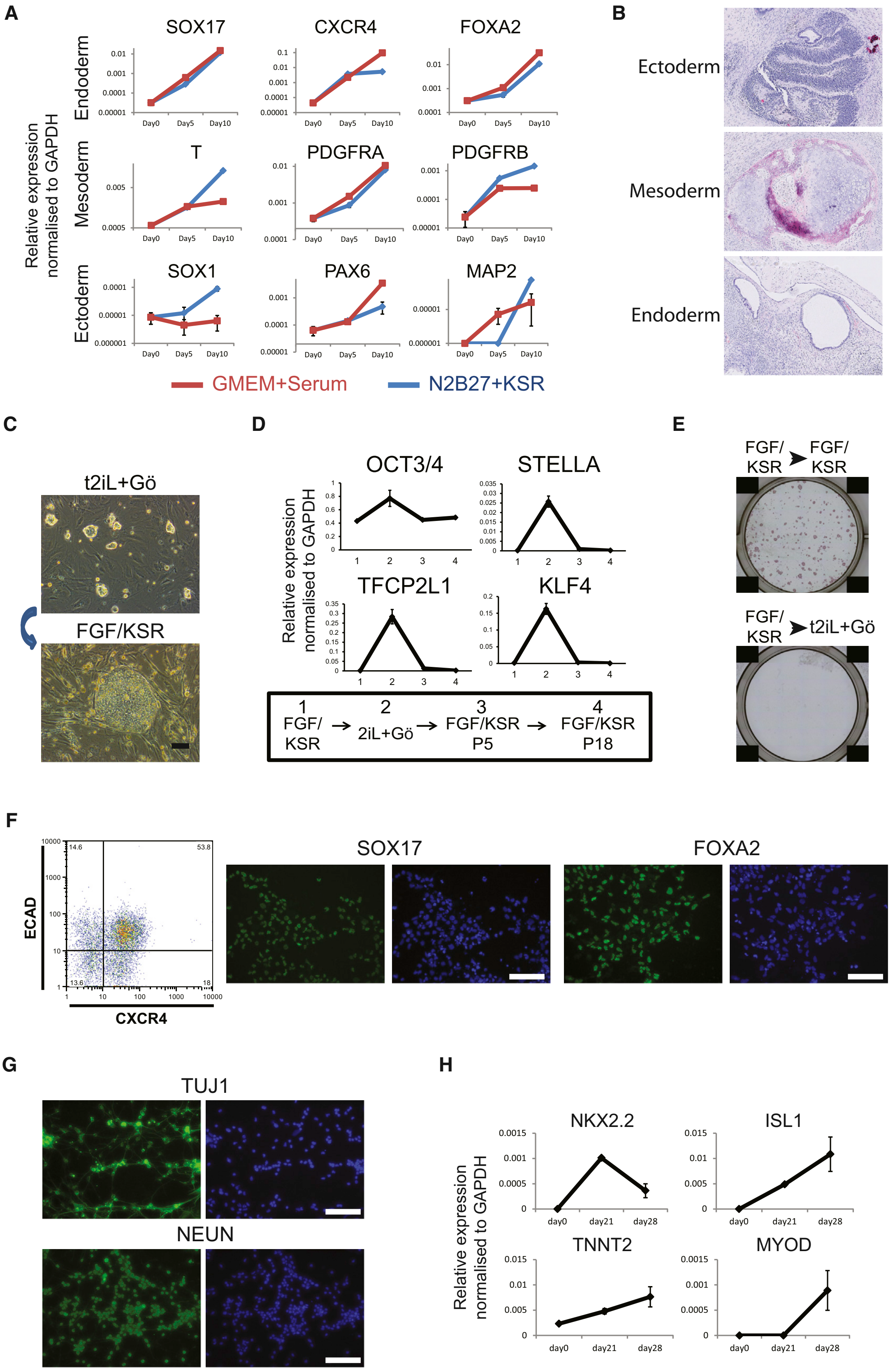

(legend on next page)

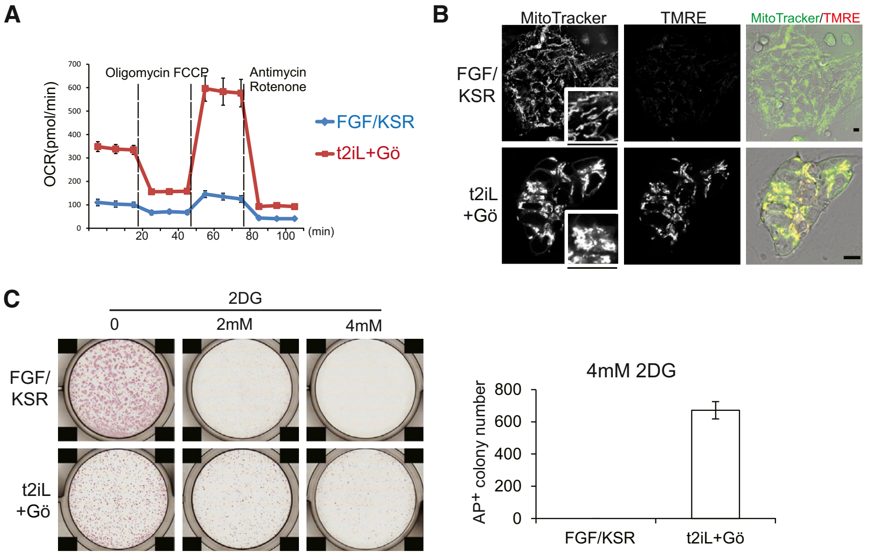

Figure 3. Mitochondrial Activity

(A) Oxygen consumption rate (OCR) measurements.

(B) Mitochondrial staining. MitoTracker is a general stain; TMRE staining is dependent on mitochondrial membrane activity. Scale bar, 10 μM; inset, 15 μM.

(C) Colony formation in 2-deoxyglucose. 3×10^4 cells were seeded in 12-well plates and were cultured for 7 days with indicated concentrations of 2-deoxyglucose (2DG).

Error bars indicate SD. See also Figure S2.

demethylation has been described for purported human naive PSC (Gafni et al., 2013), but no evidence has been provided for global changes or for demethylation of the X chromosome. The global reduction in DNA methylation in reset cells is similar in magnitude to hypomethylation in mouse ground-state ESC and in line with the demethylated status reported for the human inner cell mass (ICM) (Guo et al., 2014; Smith et al., 2014).

Transcriptome Reconfiguration

We assessed the transcriptional state of conventional human PSC, reset cells, and mouse ESC by RNA-seq. Multiple independent conventional cultures of H9 and induced PSC were analyzed alongside reset counterparts. Clustering by principal component analysis revealed mutually exclusive groups of conventional human PSC and reset cells, with distinct clusters of mouse ESC and human reset cells (Figure 5A). Much of the variation (24%) is captured in the first principal component, indicating significant correspondence between reset cells and human blastocyst ICM (Yan et al., 2013). In contrast, explanted human ICM cells propagated in FGF/KSR adopt similar expression profiles to conventional PSC cultures. Divergence with respect to the second principal component is not unexpected given that ESC bear closest resemblance to epiblast cells in the late blastocyst rather than immature ICM cells (Boroviak et al., 2014).

Inspection of genes contributing to the first two principal components confirms the influence of pluripotency factors in reset cells and of lineage specifiers in conventional PSC (Figure S4A). Analysis of an independent panel of genes selected by the International Stem Cell Initiative (Adewumi et al., 2007) shows that reset cells and ESC share similar patterns with respect to upregulation of pluripotency regulators and repression of lineage markers (Figure 5B). In contrast to conventional PSC, robust expression was observed of ground-state pluripotency regulators in both reset cells and ground-state ESC. This is accompanied by repression of lineage-specific genes. Reset cells and ESC form a distinct cluster characterized by the robust expression of naive markers including *NANOG*, *KLF4*, and *TFCP2L1*. In contrast, PSC cultured in conventional media or the 3iL formulation (Chan et al., 2013) show prominent expression of lineage markers such as AFP, Brachyury, and EOMES. Gene ontology analysis of differentially expressed genes indicated enrichment of categories representative of developmental pathways (Table S2).

We additionally compared reset and conventional PSC with NHSM or 3iL cells purported to have undergone conversion to a naive state (Chan et al., 2013; Gafni et al., 2013). To facilitate direct comparison, samples were hybridized to the identical microarray platform used in Gafni et al. (2013). We profiled an extended panel of samples, including reset cells and standard counterparts from H9 and two independent iPS cell lines, in addition to those profiled by RNA-seq above. Data from each study were normalized to conventional human embryo-derived PSC to integrate microarray and RNA-seq data sets. A significant departure from the conventional state was not apparent for cell lines propagated in alternate culture regimes, suggesting that they have not fundamentally changed from standard human PSC (Figure 5A). Analysis of data from Gafni et al. (2013) reveals

Figure 2. Differentiation

(A) Expression of lineage markers in embryoid bodies formed from reset cells in KSR or serum.

(B) Teratomas formed from reset cells in three out of ten mice.

(C) Reset cells convert to conventional PSC morphology after transfer to FGF/KSR. Bottom panel shows typical colony four passages after transfer.

(D) Downregulation of naive markers in FGF/KSR.

(E) Colony formation after transfer of reset cells into FGF/KSR for two passages. Cells plated in the presence of ROCKi.

(F) Definitive endoderm differentiation in activin and Wnt. Flow cytometry and immunostaining.

(G) Neuronal differentiation after dual inhibition of activin and BMP pathways.

(H) qRT-PCR assay of cardiac lineage markers in embryoid body outgrowths.

Scale bars: (C, F, and G) 100 μM.

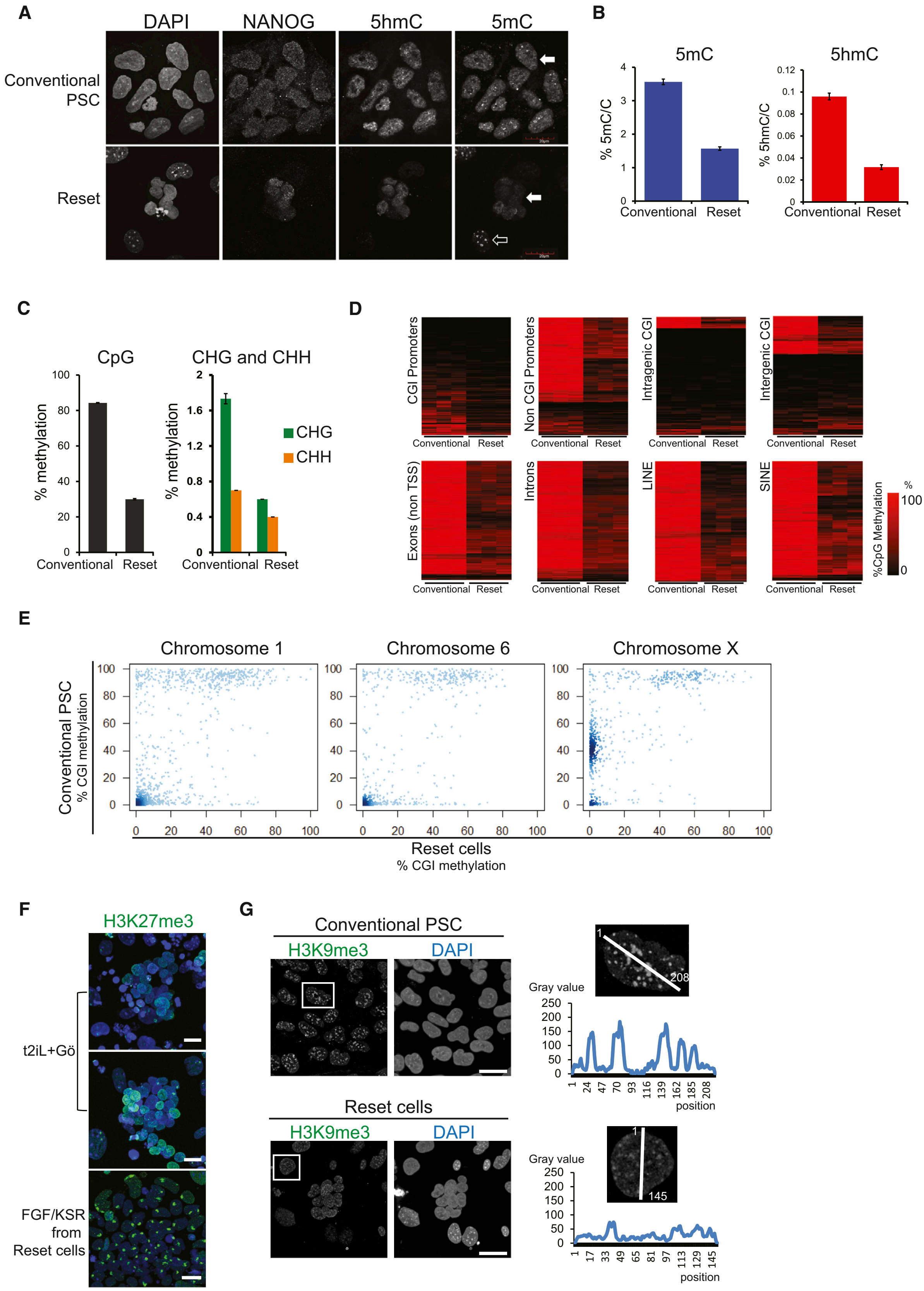

(legend on next page)

wide variation across individual lines but relatively minor divergence from other conventional human PSC cultures (Figures S5A and S5B).

Reset cells display expression patterns characteristic of ground-state ESC, in contrast to NHSM cultures where, remarkably, the expression of key pluripotency factors is often downregulated or abolished (Figure 5C). The inverse trend was evident when examining a range of lineage-specific genes, which are heavily downregulated in ESC and reset cells but display spurious expression in NHSM and 3iL cultures (Figure 5D). A number of chromatin modifiers also exhibited expression more comparable to mouse ESC for reset cells than for alternative cultures, with NHSM cells lacking expression of important epigenetic regulators such as MLL3, NCOA3, and TETs (Figure S5C). In line with the observed DNA hypomethylation in reset cells, transcripts for DNMT3B were greatly reduced and for DNMT3L increased (Neri et al., 2013).

To evaluate whether the ground-state pluripotency factors defined in mouse and upregulated in human reset cells are indeed features of human naive pluripotency, we examined two key factors, TFCP2L1 and KLF4, in human embryos. Supernumerary embryos were thawed and cultured to the expanded blastocyst stage for immunostaining (Roode et al., 2012). We detected KLF4 exclusively in the ICM (Figure 5E). TFCP2L1 signal was unambiguous in a subset of the KLF4-positive cells within the ICM. Thus, the ICM in the mature human blastocyst contains cells double positive for KLF4 and TFCP2L1 protein. Neither factor is upregulated in previously described human PSC (Figure 5C), but they are coexpressed along with NANOG in reset cells (Figure 5F), as in mouse ESC.

Executive Operation of Ground-State Transcription Factor Circuitry

To determine whether the ground-state transcription factor circuitry is functional in reset cells, we tested dependency on specific factors using RNAi. siRNA against nonessential naive pluripotency markers REX1 (ZFP42) and STELLA (DPPA3) did not impair colony formation by either reset or conventional PSC (Figure 6A). In contrast, knockdown of *KLF4* or *TFCP2L1* markedly reduced colony formation by reset cells but had little effect on conventional cultures. We then used shRNA for constitutive knockdown (Figure S6A). When *NANOG* and *KLF2* transgenes were maintained with DOX, *TFCP2L1* or *KLF4* knockdown was tolerated but cells showed reduced colony formation (Figures 6B and 6C). After DOX withdrawal, colony formation was largely abolished by knockdown in t2iL+Gö but was unaffected in FGF/KSR/ROCKi, where cells adopted flattened conventional PSC morphology (Figures 6C and S6B). *KLF4* shRNA targets the 3′UTR, and expression of human *KLF4* fully restored colony formation (Figure 6D). *TFCP2L1* shRNA targets the coding sequence, but the knockdown phenotype was partially rescued by mouse *Tfcp2l1* (Figure S6C).

Mouse ESC can withstand *Tfcp2l1* depletion due to compensation by *Esrrb* (Martello et al., 2013). Reset human cells may be sensitized due to weak expression of *ESRRB.* We therefore tested whether transgenic *ESRRB* can rescue colony formation upon *TFCP2L1* knockdown. Indeed, *ESRRB* expression rendered reset cells resistant to TFCP2L1 shRNA such that they formed multiple colonies in t2iL+Gö that had refractile domed morphology and could be expanded after passaging (Figure 6E).

These findings indicate that the self-renewal of reset human cells, but not conventional PSC, is strongly reliant on TFCP2L1 and KLF4 and furthermore point to conserved functionality of ground-state transcription factors between mouse and human, even though individual factor expression may be altered.

As a potential functional test of naive epiblast identity, we introduced reset cells into mouse preimplantation embryos and monitored development in vitro. We first used fibroblast-derived induced PSC stably transfected with CAG-Cherry before and after resetting. After morula aggregation using conventional iPSC, we did not detect any Cherry-positive cells in 37 blastocysts. In contrast, reset cells contributed to the ICM in 6 of 42 blastocysts and in some cases appeared well integrated in the epiblast compartment (Figure 6F). We repeated this test on reset cells harboring a GFP reporter and found GFP-positive cells within the ICM/epiblast in 8 of 49 blastocysts. We also assessed whether reset cells could be incorporated into the ICM by blastocyst injection. Injected embryos were cultured for 72 hr to allow hatching and primary ICM outgrowth. Of 32 injected embryos, 9 showed GFP-positive cells within the mature ICM/epiblast (Figure 6G). In contrast, none of 17 blastocysts injected with conventional Shef6 PSC showed colonization.

These data suggest that human reset cells are sufficiently similar to mouse naive cells to allow incorporation into the ICM and preimplantation epiblast.

Transient Transgenesis and Stable Resetting

We investigated the time span for resetting and found that 8 days of induction was sufficient (Figure 7A). Equivalent expression should be achievable by transient transgenesis. To identify and select reset cells, we exploited the EOS construct

Figure 4. Epigenome Analysis

(A) Immunostaining for 5mC, 5hmC, and NANOG. Conventional PSC exhibit pronounced 5mC staining (white arrow). Reset cells display reduced 5mC signal (white arrow) in contrast to feeder cells (unfilled arrow).

(B) Quantification by mass spectrometry of global 5mC and 5hmC levels.

(C) Quantitative summaries of whole-genome BS-seq data from three biological replicates.

(D) Heatmaps of methylation levels in up to 10,000 random samplings of previously classified genomic regions: CpG island (CGI) or non-CGI promoters; intragenic and intergenic CGI; exons; introns; LINEs and SINEs.

(E) Scatter plots of CGI methylation percentages on the X chromosome and autosomes.

(F) Immunostaining for H3K27me3, counterstained with DAPI. Representative fields of reset cells and after passaging in FGF/KSR.

(G) Immunostaining for H3K9me3. Intensity and distribution analysis by Image J.

Scale bars: (F and G) 20 μM. Error bars indicate SD. See also Figure S3.

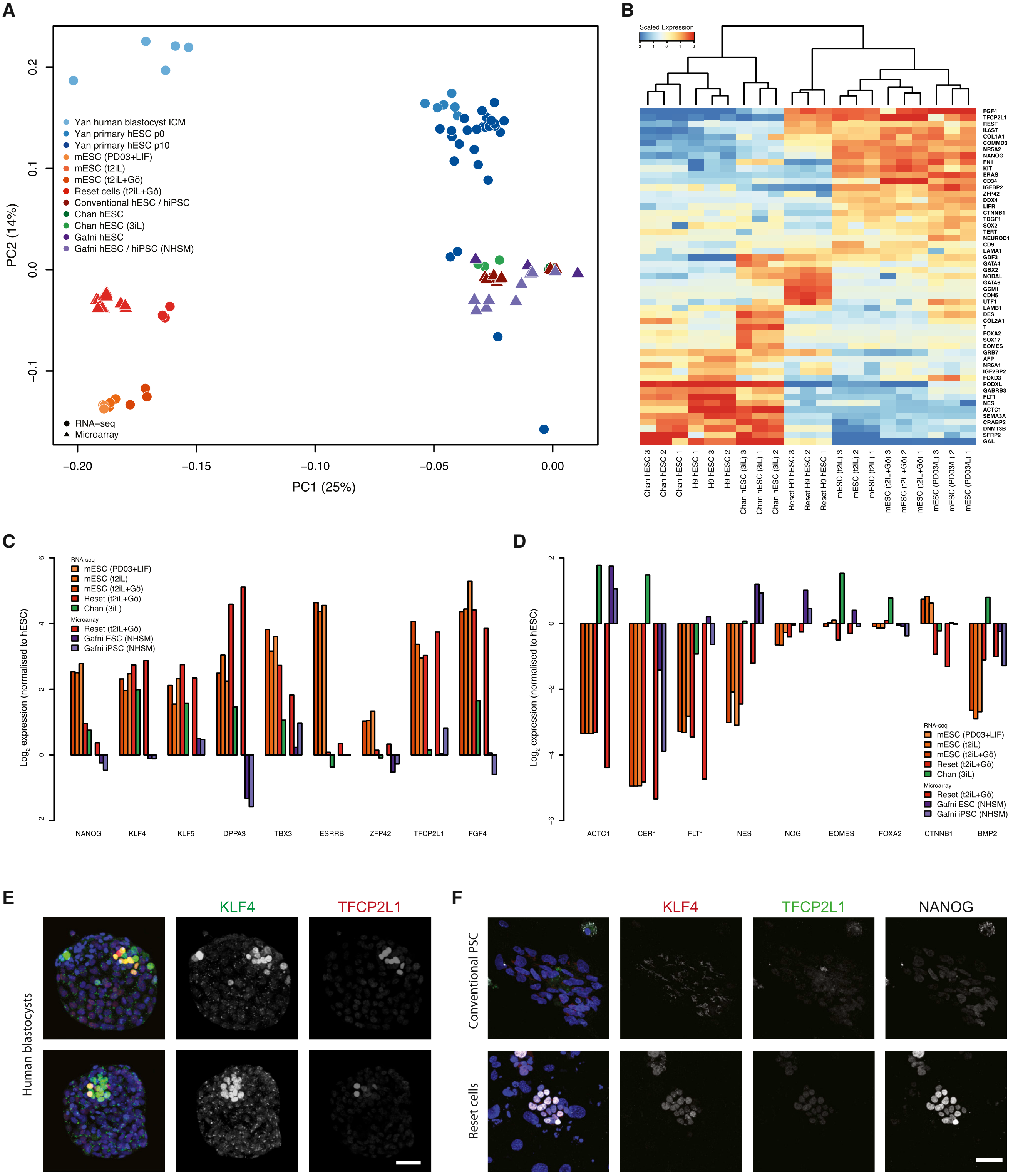

Figure 5. Comparative Expression Analysis

(A) PCA of RNA-seq and microarray data from this study with RNA-seq data from Chan et al. (2013), microarray data from Gafni et al. (2013), and single-cell RNA-seq data from Yan et al. (2013). Samples generated in this study were additionally hybridized to the identical array platform used by Gafni et al. (2013) to facilitate direct comparison. Data were normalized to conventional PSC in each study. Similar clustering is apparent using RNA-seq data alone (Figure S4B), discounting the influence of platform differences.

(legend continued on next page)

(Hotta et al., 2009) incorporating the mouse Oct4 distal enhancer active in naive, but not primed, pluripotent cells. We transfected H9 and Shef6 cells bearing an integrated PB-EOS-GFP/puro^R construct with constitutive expression plasmids for *NANOG* and *KLF2* (Figure 7B). In pilot studies, we observed that CH appeared inhibitory to the resetting process and that addition of the FGF receptor inhibitor PD17 might favor resetting. Accordingly, 2 days after transfection, medium was switched from FGF/KSR to N2B27 supplemented with PD17, PD03, and LIF. At day 4, cells were retransfected, and on day 8, medium was changed to t2iL+Gö. From day 12, several GFP-positive clusters of cells became visible and puromycin selection commenced. Cultures were heterogeneous and were passaged four times before discrete colonies were picked (Figure 7C). Seven of nine cultures showed no detectable transgene at single-copy resolution (Figure 7D). They were indistinguishable in morphology from cultures in which transgenes were detected or from reset cells generated with inducible transgenes. qRT-PCR and immunostaining confirmed sustained expression of ground-state pluripotency factors (Figures 7E and 7F). Passaging time was comparable to reset cells generated via inducible transgene expression, as was colony formation efficiency of 5%–10% without ROCKi. Colony formation in the presence of A83-01 (Figure 7G) demonstrated independence from activin/nodal, unlike other human PSC. Furthermore, knockdown of TFCP2L1 or KLF4 significantly impaired colony formation (Figure 7H), indicating dependency on ground-state transcription factors.

We conclude that the reset state can be generated without permanent genetic modification.

DISCUSSION

The postulate that a self-renewing ground state similar to rodent ESC may pertain to primates is contentious. Our findings indicate that anticipated ground-state properties may be instated in human cells following short-term expression of *NANOG* and *KLF2* transgenes. The resulting cells can be perpetuated in defined medium lacking serum products or growth factors. Feeders support attachment and growth of reset cells but are dispensable. Reset human stem cells show global changes in DNA methylation and transcription suggestive of a more primitive state. They also display altered metabolism with increased mitochondrial respiration. This constellation of features distinguishes reset cells from previous embryo-derived or induced human PSC and aligns them closer to ground-state mouse ESC. Most significantly, the unique transcription factor circuit essential for mouse ESC identity, self-renewal and pluripotency, is functionally operative in sustaining the reset human pluripotent state.

Previous claims of putative naive human PSC (Chan et al., 2013; Gafni et al., 2013; Ware et al., 2014) have employed culture media with an incoherent array of growth factors and inhibitors. One possibility is that such compound conditions may select for propagation of heterogeneous cultures comprising cells co-habiting in different phases of pluripotency, as described for mouse EpiSCs (Bernemann et al., 2011; Tsakiridis et al., 2014). Lack of enrichment for naive pluripotency factors and expression of mixed lineage markers (Figures 5C and 5D) are consistent with such an explanation.

While this study was in revision, Theunissen et al. (2014) reported that PSC cultured in a cocktail of six kinase inhibitors plus LIF and activin (6i/L/A) on feeders expressed naive features. We incorporated their data into the transcriptome meta-analysis. Use of an alternative microarray platform precludes quantitative comparison of individual genes, but PCA reveals that 6i/L/A cells are globally well separated from previously described PSC while forming a distinct cluster from reset cells (Figure S7A). Upregulation of naive markers and downregulation of lineage markers appears comparable to reset cells, but some differences are apparent in expression of epigenetic modifiers (Figure S7B). Notably, methylation regulators DNMT3a and TET1 change in opposite directions. Methylome status is not described by Theunissen et al. (2014), but they report X chromosome inactivation, in contrast to reset cells. Both X chromosomes are active in the human ICM (Okamoto et al., 2011), and the timing of inactivation is uncertain. Epiblast development is more protracted in primates than in rodents, raising the possibility that differences between reset and 6i/L/A cells may reflect successive phases of pluripotency.

Independence from Erk signaling is a hallmark of rodent naive cells that is conserved in human preimplantation epiblast (Roode et al., 2012). We used 1 μM PD03 to ensure full inhibition of the Erk pathway. In contrast, GSK3 inhibition is only partial and differs between mouse and human cells. This may reflect the balance between relief of TCF3 repressor function and activation of canonical TCF/LEF factors (Chen et al., 2013). Moreover, in mouse ESC, GSK3 inhibition acts mainly through derepression of *Esrrb* (Martello et al., 2013), but *ESRRB* is weakly expressed in reset human PSC. Poor conservation of a genomic interval in which NANOG, OCT4, SOX2, and TCF3 bind mouse *Esrrb* may underlie this lack of expression (Figure S8). ESRRB is a potent self-renewal factor in ESC (Festuccia et al., 2012; Martello et al., 2012), and its noninduction may explain the insufficiency of 2iL for human cells. Gö6983 is a broad specificity PKC inhibitor that facilitates mouse ESC self-renewal (Dutta et al., 2011). Mutation of atypical PKC iota largely recapitulates this effect in ESC (Leeb et al., 2014), whereas knockdown of aPKC iota in reset cells enhances propagation in t2iL without Gö (Figure S9). The mechanism downstream of aPKC inhibition remains to be

(B) RNA-seq meta-analysis reveals two major groups, with reset cells featuring expression patterns most similar to ESC. Values displayed correspond to the expression level in each sample scaled by the mean expression of each gene across samples.

(C) Reset cells display transcription factor hallmarks of ground-state ESC. Data normalized to expression from conventional human PSC as above.

(D) Reset cells feature downregulation of lineage markers.

(E) Immunostaining of KLF4 and TFCP2L1 in the human ICM.

(F) Coexpression of KLF4, TFCP2L1, and NANOG in reset cells.

Scale bar: (E and F) 50 μM. See also Figures S4 and S5 and Tables S2, S3 and S4.

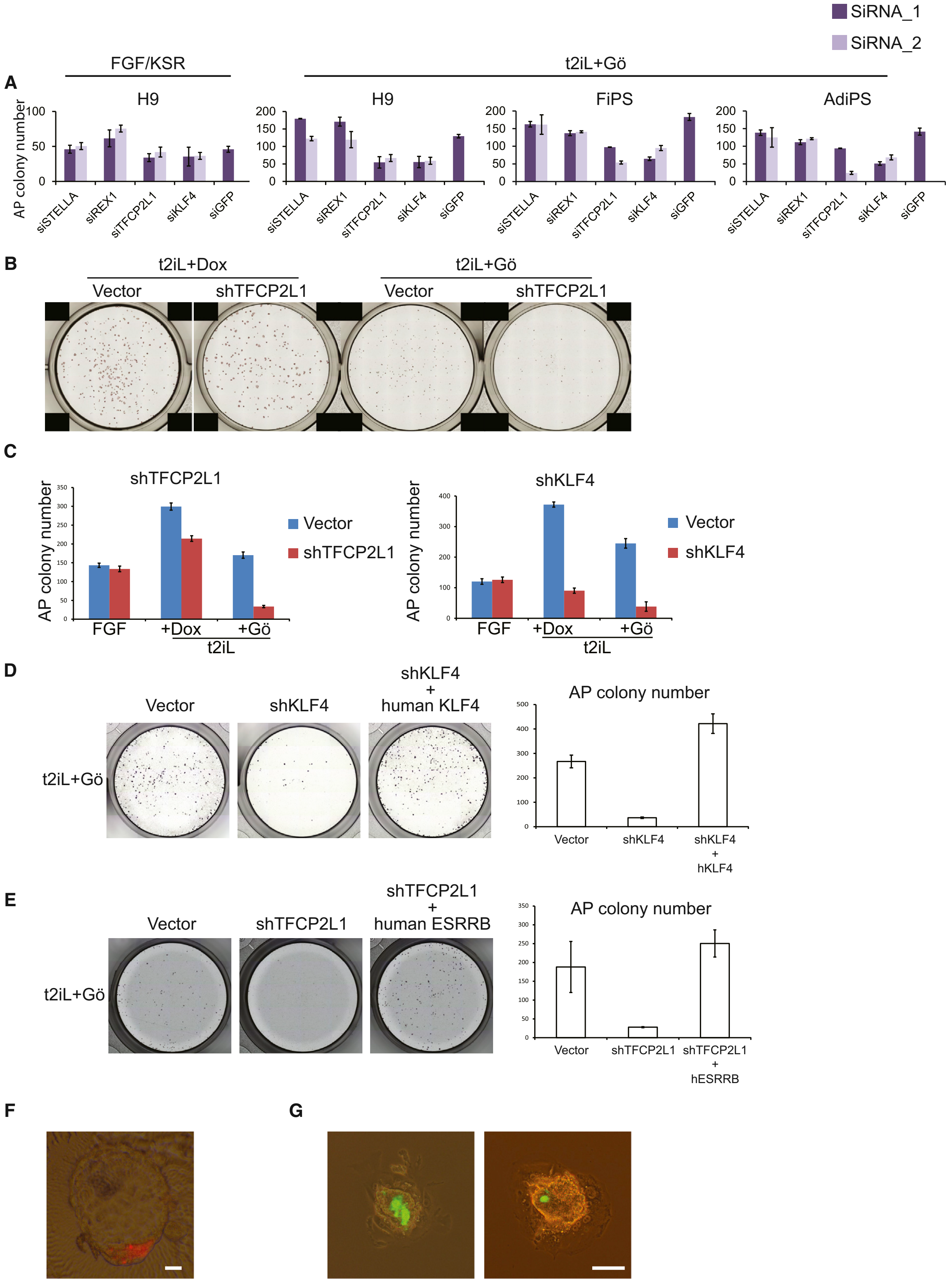

(legend on next page)

elucidated, but we speculate that differentiation may be inhibited by interfering with acquisition of epithelial polarity, an essential feature of postimplantation epiblast. On transfer to FGF/KSR, reset cells flatten and progressively adopt typical human PSC appearance and growth factor dependence. This process resembles mouse ESC to EpiSC differentiation and may mimic pre- to postimplantation epiblast progression.

Rodent ground-state ESC are distinguished by and dependent on a suite of transcription factors additional to Oct4, Sox2, and NANOG (Nichols and Smith, 2012). Each of these is individually dispensable due to overlapping functions in a flexible circuitry (Dunn et al., 2014). They are absent or very lowly expressed in EpiSC and conventional human PSC. In reset cells, all are present apart from ESRRB. Absence of ESRRB is anticipated to render the ground-state circuitry more fragile (Figure 7I). Severe compromise to self-renewal upon *KLF4* or *TFCP2L1* knockdown is in line with this prediction and provides evidence that reset human PSC are functionally governed by the rewired ground-state transcription factor circuitry. Rescue of *TFCP2L1* knockdown cells by *ESRRB* points to further functional conservation with the mouse ESC control system.

Our findings suggest that authentic ground-state pluripotent stem cells may be attainable in human, lending support to the notion of a generic naive state of pluripotency in mammals. In human, the naive-state transcription factor circuitry appears in large part to be conserved but requires greater reinforcement to be stably propagated. Disposition to collapse reflects the transient nature of naive pluripotency in the embryo (Nichols and Smith, 2009). The imperative for developmental progression may be intrinsically stronger in primates that, unlike rodents, have not evolved the facility for embryonic diapause (Nichols and Smith, 2012). Nonetheless, increased number and size of colonies under conditions of transgene induction suggest that there may be scope to refine and further improve culture conditions for human ground-state PSC.

Further evaluation of the ground-state hypothesis remains necessary. Reset cells might be considered a synthetic product of genetic intervention. Seamless derivation from human epiblast is therefore a key future landmark. Formation of primary chimeras, a powerful test of naive status and developmental potency in rodents, cannot be undertaken in human. However, the finding that reset cells can consistently be incorporated into the mouse ICM/epiblast distinguishes them from conventional human PSC or mouse EpiSC and is consistent with preimplantation identity. Interestingly, upon further culture to mimic early postimplantation stages (Bedzhov and Zernicka-Goetz, 2014), contribution of human cells to the epiblast was no longer detected. These data are preliminary but may suggest that human cells are unable to adjust to the much faster rate and/or distinct morphogenetic organization of mouse postimplantation epiblast development. Later, contribution to same-species chimeras could be explored in nonhuman primates. Perhaps the most important question, however, at least from a translational perspective, is whether rewiring transcriptional circuitry also removes epigenetic specifications. Human genetic variation notwithstanding, epigenome status may influence consistency of both undifferentiated phenotype and differentiation behavior. Low H3K9me3 and genome-wide DNA hypomethylation point to epigenome erasure in reset cells, as in early embryos. It will be of great interest to determine the precise functional impact of such epigenetic cleansing.

EXPERIMENTAL PROCEDURES

Other procedures and reagent details are provided in the Extended Experimental Procedures.

Cell Culture

Human-embryo-derived H1, H9, and Shef6 and iPS cells were maintained in conventional PSC culture conditions with FGF/KSR on feeders.

piggyBac (PB) vectors (2 μg) carrying doxycycline-inducible *KLF2* or *NANOG* coupled to Venus were cotransfected with an rtTA expression construct (2 μg) and pBase helper plasmid (4 μg) using the Neon Transfection System (Program 14; Invitrogen). Two days later, G418 was applied (100 μg/ml). After selection for 2 weeks, Venus-positive cells with leaky transgene expression were removed by flow cytometry. Transfectants were dissociated with trypsin and replated in the presence of Rho-associated kinase inhibitor (ROCKi) (Y-27632, Calbiochem) prior to addition of DOX (1 μM) on day 1. On day 2, medium was changed to N2B27 medium (Ndiff227, StemCells Inc.) with 1 μM PD0325901 (PD03), human LIF (prepared in house), CHIR99021 (3 μM; 2iL; 1 μM, t2iL), and DOX. Medium was changed daily. Cells were split every 5–7 days after dissociation with Accutase (Life Technologies). After 2 weeks, DOX was withdrawn and PKC inhibitor Gö6983 added (5 μM, Sigma-Aldrich). Cells transferred to t2iL+Gö expand slowly for the initial couple of passages after withdrawal of DOX. Subsequently, cultures in t2iL+Gö were passaged every 5–7 days using Accutase. Cells were maintained on MEF feeders throughout.

For transient expression and resetting, we established H9 and Shef6 cells with a PB-EOS-GFP/puro^R reporter (Hotta et al., 2009). This reporter is progressively silenced in conventional PSC but is re-expressed upon resetting. EOS cells were transfected with 3 μg of circular *NANOG* and *KLF2* constitutive expression plasmids. Two days later, medium was switched to N2B27 with PD173074 (PD17, 0.5 μM), PD03 (0.5 μM), and hLIF. At day 4, cells were retransfected, and on day 8, medium was changed to t2iL+Gö. Puromycin selection (0.5 μg/ml) was applied from day 12 for two passages to enrich for EOS-expressing cells. Colonies were picked on passage 4 or 5. Presence or absence of transgene vectors was assayed by genomic PCR for the CAG promoter and TaqMan Copy Number Assays against the blasticidin resistance gene with *RNase P* and *TERT* reference assays.

Figure 6. Functional Interrogation of the Reset State

(A) Colony formation after siRNA knockdown in 4,000 cells in FGF/KSR or 2,000 cells in t2iL+Gö. Colony size is variable for conventional PSC, but numbers are relatively consistent. Histogram shows mean colony counts from duplicate assays.
(B) Colony formation after shTFCP2L1 knockdown (KD) in indicated conditions.
(C) Quantification of colony formation by sh*TFCP2L1* or sh*KLF4* knockdown cells.
(D) Rescue of *KLF4* knockdown with *KLF4* transgene.
(E) Colony formation by sh*TFCP2L1* knockdown cells transfected with *ESRRB*.
(F) Morula aggregation. Six of 42 embryos aggregated with reset cells contained Cherry-positive cells, as shown. Scale bar, 20 μM.
(G) Blastocyst injection. After 72 hr, 9 of 32 embryos injected with reset cells showed GFP-positive cells in the ICM/epiblast, as shown. Scale bar, 100 μM.
Error bars indicate SD. See also Figure S6.

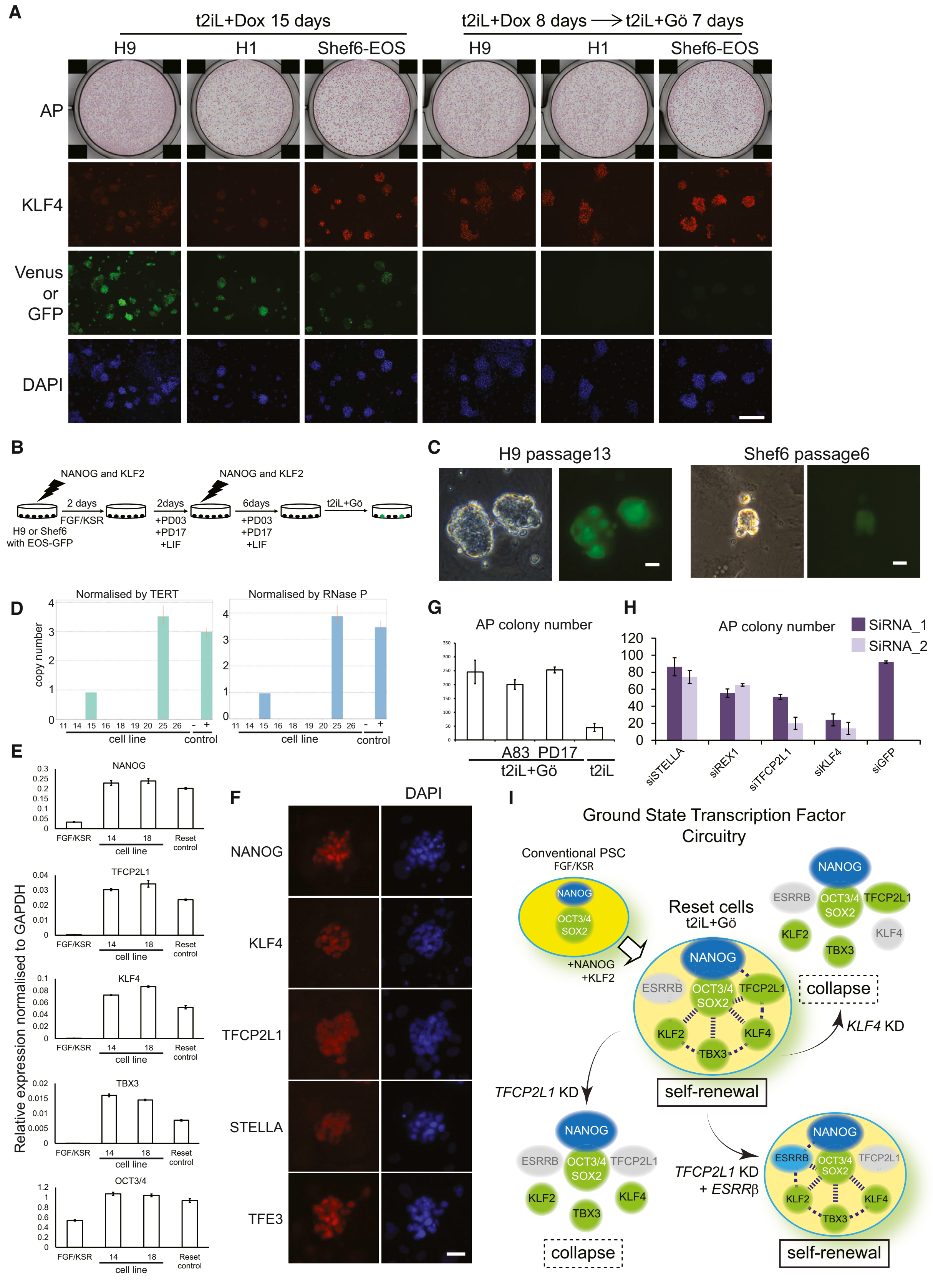

(legend on next page)

For colony-forming assays, we plated 1,000 cells per well in 24-well plates, 2,000 cells for 12-well plates, and 5,000 cells for 6-well plates. ROCKi was added for conventional but not reset cells.

For feeder-free culture, plates were coated overnight at 4°C with either diluted BD Matrigel hES-qualified Matrix (1:30) or Laminin 511-E8 (iMatrix-511; Nippi) at 0.5 mg/cm^2. Cells were dissociated in the presence of ROCKi and plated in t2iL with Gö reduced to 2 μM.

Human Embryos

Human embryo research was licensed by the UK Human Fertilization and Embryology Authority. Supernumerary embryos donated from in vitro fertilization programs with informed consent were thawed and cultured to day 7 post-fertilization. Blastocysts were fixed in 4% PFA, immunostained, and imaged as described (Roode et al., 2012).

Transcriptome Meta-Analysis

Sequencing reads were aligned to the human genome build hg19/GRCh37 with the *STAR* aligner (Dobin et al., 2013) using the two-pass method for splice junction detection (Engström et al., 2013). Transcript quantification was performed with htseq-count, part of the *HTSeq* package (Anders et al., 2014), using GENCODE v15 (Harrow et al., 2012) human gene annotation (Ensembl release 70) (Flicek et al., 2014). Sequencing reads from published RNA-seq experiments were obtained from the European Nucleotide Archive (ENA). To ensure maximal compatibility between data sets, raw counts were generated in the manner described above and all RNA-seq samples were processed together. Mouse and human samples were related via one-to-one orthologous genes annotated in Ensembl v70. Libraries were corrected for total read count using the size factors computed by the Bioconductor package *DESeq* (Anders and Huber, 2010) and were normalized for gene length to yield FPKM values. To generate expression heatmaps, FPKM values were scaled relative to the mean expression of each gene across all samples. Heatmaps include genes for which a difference in expression was observed (i.e., scaled expression > 1 or < −1 in at least one sample). Principal components were computed by singular value decomposition with the princomp function in the R *stats* package, using expression levels normalized relative to the human embryo-derived PSC samples in each study.

Affymetrix Human Gene Array 1.0 ST arrays were processed with the *oligo* Bioconductor package (Carvalho and Irizarry, 2010) to summarize probe-set transcript clusters. Microarray data from this study were normalized together with those from Gafni et al. (2013) using the robust multi-array average (RMA) method (Irizarry et al., 2003) applied through the *oligo* package. Principal components were calculated from the centered and scaled expression covariance matrix by singular value decomposition, computed by the prcomp function in the R *stats* package. Transcript clusters were associated with targeted genes based on GENCODE v15 human genome annotation (Ensembl release 70). Where multiple probe sets for a given gene were present on the array, these were summarized using the maximal expression value. Expression data for heatmaps were scaled relative to the mean expression of each gene across all samples. Affymetrix PrimeView arrays from Theunissen et al. (2014) were normalized with the RMA algorithm implemented in the *affy* Bioconductor package using a modified CDF environment to annotate ERCC control probes.

RNA-seq data were cross-referenced with the microarray data, restricting the analysis to the genes interrogated by each array design. To account for technical differences between experiments and platforms, expression levels were computed relative to the human embryo-derived PSC samples from each study. These values were used as the basis for global PCA and comparative analysis of marker genes.

Embryo Chimeras

Cells were stably transfected with PB-Cherry or PB-GFP reporters. Five to ten cells were aggregated with eight-cell stage mouse embryos and imaging performed 48 hr later at the expanded blastocyst stage. Alternatively, 8–12 cells were injected per blastocyst (E3.5) with imaging of hatched outgrowths 72 hr later. In some cases, outgrowths were cultured further to form egg cylinder-like rosettes (Bedzhov and Zernicka-Goetz, 2014).

ACCESSION NUMBERS

RNA-seq and microarray data are available in ArrayExpress under accessions E-MTAB-2857 and E-MTAB-2856. BS-seq data are available in GEO under accession GSE60945.

SUPPLEMENTAL INFORMATION

Supplemental Information includes Extended Experimental Procedures, nine figures, and four tables and can be found with this article online at http://dx.doi.org/10.1016/j.cell.2014.08.029.

AUTHOR CONTRIBUTIONS

G.G., Y.T., and A.S. conceived the approach. Y.T. designed, performed, and interpreted cell culture experiments with contributions from G.G. and J.C. J.N. carried out human embryo analyses. R.L. performed integrated transcriptome analyses. W.M. produced teratomas and chimaeras. G.F. carried out BS-seq with bioinformatics analyses by F.K. D.O. performed mass spectrometry, and F.S. contributed to methylome studies, overseen by W.R. P.B. generated sequencing libraries, carried out RNA-seq and microarray analyses, and oversaw computational work. Y.T., P.B., and A.S. wrote the paper.

ACKNOWLEDGMENTS

We thank Hitoshi Niwa for DOX-regulatable PB vector, Noemi Fusaki for Sendai virus reprogramming constructs, and Ivan Damjanov for teratoma assessment. We also thank Sally Marchant (Barts Hospital) and couples who donated embryos. Illumina sequencing and microarray hybridization were carried out by Bettina Haase and Jelena Pistolic at the EMBL Genomics Core Facility. CytoScan HD array analysis was performed by Rehannah Borup at the Center for Genomic Medicine, Copenhagen University Hospital. Sam Myers and Masayo Fujiwara provided technical assistance, and Peter Humphreys performed

Figure 7. Resetting by Transient Transgenesis

(A) Time span for resetting with inducible *NANOG* and *KLF2*. At day 8, cultures were replated in triplicate with or without DOX. Colonies were analyzed at day 15. Few colonies are obtained with DOX exposure <6 days. Reporter expression in Shef6-EOS cells was assessed by GFP after DOX withdrawal. Exposure time was constant for all images, yielding lower EOS-GFP signal relative to hCMV-Venus. Scale bar, 100 μM.
(B) Scheme for generation of reset cells by transient transfection.
(C) Phase contrast and fluorescence images of reset cells generated by transient transfection of H9 and Shef6 PSC.
(D) Detection of transgene-free cultures by TaqMan copy number assay. Cells with (+) or without (−) a blasticidin transgene provide controls.
(E) qRT-PCR assay for transcription factor expression in expanded transgene-free reset cells.
(F) Immunofluorescence staining of expanded transgene-free reset cells.
(G) Colony-forming assays on transgene-free reset cells seeded in the indicated conditions without ROCKi.
(H) Colony formation after siRNA knockdown in transgene-free reset cells. Histogram shows mean colony counts from duplicate assays.
(I) ESC express general pluripotency factors Oct4 and Sox2 plus an interconnected transcription factor circuitry that sustains self-renewal. Resetting induces expression of these factors in human PSC apart from *ESRRB*. Self-renewal is less robust in human, and knockdown of single components, TFCP2L1, or KLF4 causes collapse.
Error bars indicate SD.

confocal imaging. This research was supported by the UK Medical Research Council, the Japan Science and Technology agency (JST, PRESTO), the Genome Biology Unit of the European Molecular Biology Laboratory, European Commission projects PluriMes, BetaCellTherapy, EpiGeneSys, and Blueprint, and the Wellcome Trust. Y.T. was a University of Cambridge Herchel Smith Fellow. A.S. is a Medical Research Council Professor.

Received: April 7, 2014
Revised: July 30, 2014
Accepted: August 22, 2014
Published: September 11, 2014

REFERENCES

Adewumi, O., Aflatoonian, B., Ahrlund-Richter, L., Amit, M., Andrews, P.W., Beighton, G., Bello, P.A., Benvenisty, N., Berry, L.S., Bevan, S., et al.; International Stem Cell Initiative (2007). Characterization of human embryonic stem cell lines by the International Stem Cell Initiative. Nat. Biotechnol. *25*, 803–816.

Amit, M., Carpenter, M.K., Inokuma, M.S., Chiu, C.P., Harris, C.P., Waknitz, M.A., Itskovitz-Eldor, J., and Thomson, J.A. (2000). Clonally derived human embryonic stem cell lines maintain pluripotency and proliferative potential for prolonged periods of culture. Dev. Biol. *227*, 271–278.

Anders, S., and Huber, W. (2010). Differential expression analysis for sequence count data. Genome Biol. *11*, R106.

Anders, S., Pyl, P.T., and Huber, W. (2014). HTSeq — A Python framework to work with high-throughput sequencing data. bioRxiv. Published online February 20, 2014. http://dx.doi.org/10.1101/002824.

Bedzhov, I., and Zernicka-Goetz, M. (2014). Self-organizing properties of mouse pluripotent cells initiate morphogenesis upon implantation. Cell *156*, 1032–1044.

Bernemann, C., Greber, B., Ko, K., Sterneckert, J., Han, D.W., Araúzo-Bravo, M.J., and Schöler, H.R. (2011). Distinct developmental ground states of epiblast stem cell lines determine different pluripotency features. Stem Cells *29*, 1496–1503.

Betschinger, J., Nichols, J., Dietmann, S., Corrin, P.D., Paddison, P.J., and Smith, A. (2013). Exit from pluripotency is gated by intracellular redistribution of the bHLH transcription factor Tfe3. Cell *153*, 335–347.

Boroviak, T., Loos, R., Bertone, P., Smith, A., and Nichols, J. (2014). The ability of inner-cell-mass cells to self-renew as embryonic stem cells is acquired following epiblast specification. Nat. Cell Biol. *16*, 516–528.

Brons, I.G., Smithers, L.E., Trotter, M.W., Rugg-Gunn, P., Sun, B., Chuva de Sousa Lopes, S.M., Howlett, S.K., Clarkson, A., Ahrlund-Richter, L., Pedersen, R.A., and Vallier, L. (2007). Derivation of pluripotent epiblast stem cells from mammalian embryos. Nature *448*, 191–195.

Carvalho, B.S., and Irizarry, R.A. (2010). A framework for oligonucleotide microarray preprocessing. Bioinformatics *26*, 2363–2367.

Chambers, S.M., Fasano, C.A., Papapetrou, E.P., Tomishima, M., Sadelain, M., and Studer, L. (2009). Highly efficient neural conversion of human ES and iPS cells by dual inhibition of SMAD signaling. Nat. Biotechnol. *27*, 275–280.

Chan, Y.S., Göke, J., Ng, J.H., Lu, X., Gonzales, K.A., Tan, C.P., Tng, W.Q., Hong, Z.Z., Lim, Y.S., and Ng, H.H. (2013). Induction of a human pluripotent state with distinct regulatory circuitry that resembles preimplantation epiblast. Cell Stem Cell *13*, 663–675.

Chen, Y., Blair, K., and Smith, A. (2013). Robust self-renewal of rat embryonic stem cells requires fine-tuning of glycogen synthase kinase-3 inhibition. Stem Cell Rev. *1*, 209–217.

Dobin, A., Davis, C.A., Schlesinger, F., Drenkow, J., Zaleski, C., Jha, S., Batut, P., Chaisson, M., and Gingeras, T.R. (2013). STAR: ultrafast universal RNA-seq aligner. Bioinformatics *29*, 15–21.

Dunn, S.J., Martello, G., Yordanov, B., Emmott, S., and Smith, A.G. (2014). Defining an essential transcription factor program for naïve pluripotency. Science *344*, 1156–1160.

Dutta, D., Ray, S., Home, P., Larson, M., Wolfe, M.W., and Paul, S. (2011). Self-renewal versus lineage commitment of embryonic stem cells: protein kinase C signaling shifts the balance. Stem Cells *29*, 618–628.

Engström, P.G., Steijger, T., Sipos, B., Grant, G.R., Kahles, A., Rätsch, G., Goldman, N., Hubbard, T.J., Harrow, J., Guigó, R., and Bertone, P.; RGASP Consortium (2013). Systematic evaluation of spliced alignment programs for RNA-seq data. Nat. Methods *10*, 1185–1191.

Festuccia, N., Osorno, R., Halbritter, F., Karwacki-Neisius, V., Navarro, P., Colby, D., Wong, F., Yates, A., Tomlinson, S.R., and Chambers, I. (2012). Esrrb is a direct Nanog target gene that can substitute for Nanog function in pluripotent cells. Cell Stem Cell *11*, 477–490.

Ficz, G., Hore, T.A., Santos, F., Lee, H.J., Dean, W., Arand, J., Krueger, F., Oxley, D., Paul, Y.L., Walter, J., et al. (2013). FGF signaling inhibition in ESCs drives rapid genome-wide demethylation to the epigenetic ground state of pluripotency. Cell Stem Cell *13*, 351–359.

Flicek, P., Amode, M.R., Barrell, D., Beal, K., Billis, K., Brent, S., Carvalho-Silva, D., Clapham, P., Coates, G., Fitzgerald, S., et al. (2014). Ensembl 2014. Nucleic Acids Res. *42* (Database issue), D749–D755.

Gafni, O., Weinberger, L., Mansour, A.A., Manor, Y.S., Chomsky, E., Ben-Yosef, D., Kalma, Y., Viukov, S., Maza, I., Zviran, A., et al. (2013). Derivation of novel human ground state naive pluripotent stem cells. Nature *504*, 282–286.

Guo, G., Yang, J., Nichols, J., Hall, J.S., Eyres, I., Mansfield, W., and Smith, A. (2009). Klf4 reverts developmentally programmed restriction of ground state pluripotency. Development *136*, 1063–1069.

Guo, H., Zhu, P., Yan, L., Li, R., Hu, B., Lian, Y., Yan, J., Ren, X., Lin, S., Li, J., et al. (2014). The DNA methylation landscape of human early embryos. Nature *511*, 606–610.

Habibi, E., Brinkman, A.B., Arand, J., Kroeze, L.I., Kerstens, H.H., Matarese, F., Lepikhov, K., Gut, M., Brun-Heath, I., Hubner, N.C., et al. (2013). Whole-genome bisulfite sequencing of two distinct interconvertible DNA methylomes of mouse embryonic stem cells. Cell Stem Cell *13*, 360–369.

Hall, J., Guo, G., Wray, J., Eyres, I., Nichols, J., Grotewold, L., Morfopoulou, S., Humphreys, P., Mansfield, W., Walker, R., et al. (2009). Oct4 and LIF/Stat3 additively induce Krüppel factors to sustain embryonic stem cell self-renewal. Cell Stem Cell *5*, 597–609.

Hanna, J., Markoulaki, S., Mitalipova, M., Cheng, A.W., Cassady, J.P., Staerk, J., Carey, B.W., Lengner, C.J., Foreman, R., Love, J., et al. (2009). Metastable pluripotent states in NOD-mouse-derived ESCs. Cell Stem Cell *4*, 513–524.

Hanna, J., Cheng, A.W., Saha, K., Kim, J., Lengner, C.J., Soldner, F., Cassady, J.P., Muffat, J., Carey, B.W., and Jaenisch, R. (2010). Human embryonic stem cells with biological and epigenetic characteristics similar to those of mouse ESCs. Proc. Natl. Acad. Sci. USA *107*, 9222–9227.

Harrow, J., Frankish, A., Gonzalez, J.M., Tapanari, E., Diekhans, M., Kokocinski, F., Aken, B.L., Barrell, D., Zadissa, A., Searle, S., et al. (2012). GENCODE: the reference human genome annotation for The ENCODE Project. Genome Res. *22*, 1760–1774.

Hotta, A., Cheung, A.Y., Farra, N., Garcha, K., Chang, W.Y., Pasceri, P., Stanford, W.L., and Ellis, J. (2009). EOS lentiviral vector selection system for human induced pluripotent stem cells. Nat. Protoc. *4*, 1828–1844.

Irizarry, R.A., Hobbs, B., Collin, F., Beazer-Barclay, Y.D., Antonellis, K.J., Scherf, U., and Speed, T.P. (2003). Exploration, normalization, and summaries of high density oligonucleotide array probe level data. Biostatistics *4*, 249–264.

Ivanova, N., Dobrin, R., Lu, R., Kotenko, I., Levorse, J., DeCoste, C., Schafer, X., Lun, Y., and Lemischka, I.R. (2006). Dissecting self-renewal in stem cells with RNA interference. Nature *442*, 533–538.

Kojima, Y., Kaufman-Francis, K., Studdert, J.B., Steiner, K.A., Power, M.D., Loebel, D.A., Jones, V., Hor, A., de Alencastro, G., Logan, G.J., et al. (2014). The transcriptional and functional properties of mouse epiblast stem cells resemble the anterior primitive streak. Cell Stem Cell *14*, 107–120.

Kroon, E., Martinson, L.A., Kadoya, K., Bang, A.G., Kelly, O.G., Eliazer, S., Young, H., Richardson, M., Smart, N.G., Cunningham, J., et al. (2008).

Pancreatic endoderm derived from human embryonic stem cells generates glucose-responsive insulin-secreting cells in vivo. Nat. Biotechnol. *26*, 443–452.

Leeb, M., Dietmann, S., Paramor, M., Niwa, H., and Smith, A. (2014). Genetic exploration of the exit from self-renewal using haploid embryonic stem cells. Cell Stem Cell *14*, 385–393.

Leitch, H.G., McEwen, K.R., Turp, A., Encheva, V., Carroll, T., Grabole, N., Mansfield, W., Nashun, B., Knezovich, J.G., Smith, A., et al. (2013). Naive pluripotency is associated with global DNA hypomethylation. Nat. Struct. Mol. Biol. *20*, 311–316.

Martello, G., Sugimoto, T., Diamanti, E., Joshi, A., Hannah, R., Ohtsuka, S., Göttgens, B., Niwa, H., and Smith, A. (2012). Esrrb is a pivotal target of the Gsk3/Tcf3 axis regulating embryonic stem cell self-renewal. Cell Stem Cell *11*, 491–504.

Martello, G., Bertone, P., and Smith, A. (2013). Identification of the missing pluripotency mediator downstream of leukaemia inhibitory factor. EMBO J. *32*, 2561–2574.

Meek, S., Wei, J., Sutherland, L., Nilges, B., Buehr, M., Tomlinson, S.R., Thomson, A.J., and Burdon, T. (2013). Tuning of β-catenin activity is required to stabilize self-renewal of rat embryonic stem cells. Stem Cells *31*, 2104–2115.

Nakagawa, M., Taniguchi, Y., Senda, S., Takizawa, N., Ichisaka, T., Asano, K., Morizane, A., Doi, D., Takahashi, J., Nishizawa, M., et al. (2014). A novel efficient feeder-free culture system for the derivation of human induced pluripotent stem cells. Sci. Rep. *4*, 3594.

Neri, F., Krepelova, A., Incarnato, D., Maldotti, M., Parlato, C., Galvagni, F., Matarese, F., Stunnenberg, H.G., and Oliviero, S. (2013). Dnmt3L antagonizes DNA methylation at bivalent promoters and favors DNA methylation at gene bodies in ESCs. Cell *155*, 121–134.

Nichols, J., and Smith, A. (2009). Naive and primed pluripotent states. Cell Stem Cell *4*, 487–492.

Nichols, J., and Smith, A. (2012). Pluripotency in the embryo and in culture. Cold Spring Harb. Perspect. Biol. *4*, a008128.

Niwa, H., Ogawa, K., Shimosato, D., and Adachi, K. (2009). A parallel circuit of LIF signalling pathways maintains pluripotency of mouse ES cells. Nature *460*, 118–122.

Okamoto, I., Patrat, C., Thépot, D., Peynot, N., Fauque, P., Daniel, N., Diabangouaya, P., Wolf, J.P., Renard, J.P., Duranthon, V., and Heard, E. (2011). Eutherian mammals use diverse strategies to initiate X-chromosome inactivation during development. Nature *472*, 370–374.

Roode, M., Blair, K., Snell, P., Elder, K., Marchant, S., Smith, A., and Nichols, J. (2012). Human hypoblast formation is not dependent on FGF signalling. Dev. Biol. *361*, 358–363.

Silva, S.S., Rowntree, R.K., Mekhoubad, S., and Lee, J.T. (2008). X-chromosome inactivation and epigenetic fluidity in human embryonic stem cells. Proc. Natl. Acad. Sci. USA *105*, 4820–4825.

Silva, J., Nichols, J., Theunissen, T.W., Guo, G., van Oosten, A.L., Barrandon, O., Wray, J., Yamanaka, S., Chambers, I., and Smith, A. (2009). Nanog is the gateway to the pluripotent ground state. Cell *138*, 722–737.

Smith, Z.D., Chan, M.M., Humm, K.C., Karnik, R., Mekhoubad, S., Regev, A., Eggan, K., and Meissner, A. (2014). DNA methylation dynamics of the human preimplantation embryo. Nature *511*, 611–615.

Tesar, P.J., Chenoweth, J.G., Brook, F.A., Davies, T.J., Evans, E.P., Mack, D.L., Gardner, R.L., and McKay, R.D. (2007). New cell lines from mouse epiblast share defining features with human embryonic stem cells. Nature *448*, 196–199.

Theunissen, T.W., Powell, B.E., Wang, H., Mitalipova, M., Faddah, D.A., Reddy, J., Fan, Z.P., Maetzel, D., Ganz, K., Shi, L., et al. (2014). Systematic identification of defined conditions for induction and maintenance of naive human pluripotency. Cell Stem Cell. Published online July 24, 2014. http://dx.doi.org/10.1016/j.stem.2014.07.002.

Tomoda, K., Takahashi, K., Leung, K., Okada, A., Narita, M., Yamada, N.A., Eilertson, K.E., Tsang, P., Baba, S., White, M.P., et al. (2012). Derivation conditions impact X-inactivation status in female human induced pluripotent stem cells. Cell Stem Cell *11*, 91–99.

Tsakiridis, A., Huang, Y., Blin, G., Skylaki, S., Wymeersch, F., Osorno, R., Economou, C., Karagianni, E., Zhao, S., Lowell, S., and Wilson, V. (2014). Distinct Wnt-driven primitive streak-like populations reflect in vivo lineage precursors. Development *141*, 1209–1221.

Vallier, L., Alexander, M., and Pedersen, R.A. (2005). Activin/Nodal and FGF pathways cooperate to maintain pluripotency of human embryonic stem cells. J. Cell Sci. *118*, 4495–4509.

Wang, W., Yang, J., Liu, H., Lu, D., Chen, X., Zenonos, Z., Campos, L.S., Rad, R., Guo, G., Zhang, S., et al. (2011). Rapid and efficient reprogramming of somatic cells to induced pluripotent stem cells by retinoic acid receptor gamma and liver receptor homolog 1. Proc. Natl. Acad. Sci. USA *108*, 18283–18288.

Ware, C.B., Nelson, A.M., Mecham, B., Hesson, J., Zhou, W., Jonlin, E.C., Jimenez-Caliani, A.J., Deng, X., Cavanaugh, C., Cook, S., et al. (2014). Derivation of naive human embryonic stem cells. Proc. Natl. Acad. Sci. USA *111*, 4484–4489.

Watanabe, K., Ueno, M., Kamiya, D., Nishiyama, A., Matsumura, M., Wataya, T., Takahashi, J.B., Nishikawa, S., Nishikawa, S., Muguruma, K., and Sasai, Y. (2007). A ROCK inhibitor permits survival of dissociated human embryonic stem cells. Nat. Biotechnol. *25*, 681–686.

Wray, J., Kalkan, T., and Smith, A.G. (2010). The ground state of pluripotency. Biochem. Soc. Trans. *38*, 1027–1032.

Yan, L., Yang, M., Guo, H., Yang, L., Wu, J., Li, R., Liu, P., Lian, Y., Zheng, X., Yan, J., et al. (2013). Single-cell RNA-Seq profiling of human preimplantation embryos and embryonic stem cells. Nat. Struct. Mol. Biol. *20*, 1131–1139.

Ye, S., Li, P., Tong, C., and Ying, Q.L. (2013). Embryonic stem cell self-renewal pathways converge on the transcription factor Tfcp2l1. EMBO J. *32*, 2548–2560.

Ying, Q.L., Wray, J., Nichols, J., Batlle-Morera, L., Doble, B., Woodgett, J., Cohen, P., and Smith, A. (2008). The ground state of embryonic stem cell self-renewal. Nature *453*, 519–523.

Zhou, W., Choi, M., Margineantu, D., Margaretha, L., Hesson, J., Cavanaugh, C., Blau, C.A., Horwitz, M.S., Hockenbery, D., Ware, C., and Ruohola-Baker, H. (2012). HIF1α induced switch from bivalent to exclusively glycolytic metabolism during ESC-to-EpiSC/hESC transition. EMBO J. *31*, 2103–2116.

EXTENDED EXPERIMENTAL PROCEDURES

Cell Lines and Culture Details

Human embryo-derived cells used were H1, H9 (WiCell Research Institute) (Thomson et al., 1998) and Shef6 (Aflatoonian et al., 2010). Human iPS cells were generated from adult keratinocytes (Invitrogen), fibroblasts (Invitrogen), or adipose-derived stem cells (Invitrogen). Conventional PSC medium (FGF/KSR) comprised DMEM/F-12 (Sigma-Aldrich) with 10 ng/ml bFGF (prepared in-house) and 20% KSR (Invitrogen) supplemented with 100 mM 2-mercaptoethanol (2ME) (Sigma-Aldrich M7522), MEM non-essential amino acids (NEAA) (Invitrogen 11140050), 2 mM L-glutamine (Invitrogen, cat. 25030024). Cultures were passaged every five to seven days as small clumps by dissociation with 0.025% Trypsin, 1 mg/ml Collagenase IV (Invitrogen 17104-019), KSR (final 20%), 1mM $CaCl_2$. Throughout this study cells were maintained on irradiated mouse embryonic fibroblasts (MEF) in 5% oxygen.

Colony assays were scored by fixing and staining for alkaline phosphatase, typically 7 days after plating. For proliferation assays triplicate wells were seeded with $1x10^5$ cells. Cultures were dissociated, counted and passaged every 6 days, reseeding at the starting density.

Mouse ES cells were cultured on feeders in PD03 + LIF, t2iL or t2iL+Gö to produce RNA for comparative transcriptome analyses.

In Vitro Differentiation

For embryoid body formation, 10,000 reset cells dissociated with Accutase were plated per well of a PrimeSurface 96V cell plate (Sumitomo Bakelite MS-9096V) in two differentiation media: GMEM (Sigma G5154) with L-glutamine, pyruvate, 2ME and NEAA plus 10% serum; or N2B27 with 10% KSR. Medium was changed every second day. RNA was prepared from cells harvested at days 0, 5, and 10. For endoderm differentiation, reset cells were seeded on Matrigel (growth factor reduced, BD, 356230) coated plates in mTeSR medium for one week and then transferred into RPMI with 100 ng/ml activin A (prepared in-house) and 25 ng/ml mouse Wnt3A (R&D Systems) (Kroon et al., 2008). The following day medium was changed to RPMI with 100ng activin A and 0.2% serum. Flow cytometry for CXCR4 and E-cadherin was performed at day 3 and immunofluorescence for SOX17 and FOXA2 was performed at day 4. For cardiomyocyte differentiation, reset cells were passaged in FGF/KSR medium on MEF feeder cells then 10,000 cells were plated per V-bottom well in MEF-conditioned FGF/KSR medium containing 10 μM ROCKi. At day 3 medium was changed to DMEM/F-12 with 20% FBS, L-glutamine, MEM amino acids (Life Technologies 11130), 2-mercaptoethanol, and 50 μg/ml ascorbic acid (Moretti et al., 2010). At day 7, aggregates were seeded on gelatin-coated wells. Differentiation medium was changed every two days. Beating foci appeared from 21 days. For neural induction, reset cells were first cultured in FGF/KSR for a minimum of 6 days. Dissociated cells were then seeded on Matrigel (growth factor reduced, BD 356230) coated plates in mTeSR for two days before medium was changed to Ndiff227 (StemCells Inc.) with 10 ng/ml FGF, 20 μM SB431542 and 260 ng/ml Noggin (R&D) (Chambers et al., 2009). At day 5, medium was changed to Ndiff227 with 10 ng/ml FGF, 20 μM SB431542. At day 10, cells were fixed and stained for TUJ1 and NEUN.

Teratoma Formation

Studies were carried out in a designated facility under licenses granted by the UK Home Office. Approximately 10^5 cells were injected under renal capsules of NOD/SCID mice. After 12 weeks teratomas were excised, fixed with 4% PFA, sectioned and stained with hematoxylin and eosin.

Karyotype Analysis

KaryoMAX (Invitrogen, final concentration 0.06 μg/ml) was added to culture medium and cells incubated for 6 hr at 37°C. Cells in suspension were collected and washed in PBS, then incubated in 5ml of a pre-warmed (37°C), 0.075M potassium chloride for 10 min (37°C). After centrifugation, 4ml fixative (3:1 methanol:acetic acid) was added. This fixation step was repeated twice. Fixed samples were analyzed as G-banded karyotypes at the Medical Genetics Laboratories, Cambridge University Hospitals NHS Foundation Trust.

Genome Integrity

The CytoScan HD Array platform (Affymetrix) was used to screen for chromosomal abnormalities. Genomic DNA was isolated by QIAamp mini kit (QIAGEN 51304) and analysis performed at Center for Genomic Medicine, Copenhagen University Hospital.

Reverse Transcription Quantitative Real-Time PCR

Total RNA was isolated using the RNeasy kit (QIAGEN) and complementary DNA (cDNA) made from 1000ng of RNA using SuperScript III (Invitrogen) and oligo-dT primers. For real-time PCR, we used TaqMan Fast Universal Master Mix and TaqMan probes (Applied Biosystems) or the Universal Probe Library (UPL, Roche) system. Two or three technical replicates and at least two independent cultures were assayed for all quantitative PCR reactions. An endogenous control (Human GAPD, Applied Biosystems 4352934E) was used to normalize expression. Primers and UPL probe numbers are listed below.

Taqman	
**Gene**	**Taqman probe**
NANOG	Hs02387400_g1
KLF2	Hs00360439_g1
OCT3/4	Hs03005111_g1
TBX3	Hs00195612_m1
REX1	Hs00399279_m1
TFCP2L1	Hs00232708_m1
KLF4	Hs00358836_m1
GBX2	Hs00230965_m1
SALL4	Hs00360675_m1
ESRRB	Hs01584024_m1
SOX17	Hs00751752_s1
CXCR4	Hs00607978_s1
FOXA2	Hs00232764_m1
T	Hs00610080_m1
PDGFRA	Hs00998018_m1
PDGFRB	Hs01019589_m1
SOX1	Hs01057642_s1
PAX6	Hs01088112_m1
MAP2	Hs00159041_m1
NKX2-2	Hs00159616_m1
TNNT2	Hs00165960_m1
MYOCD	Hs00538071_m1
PRKCZ	Hs00177051_m1
PRKCI	Hs00995854_g1
hCMV1NANOG	Custom TaqMan Assays*
hCMV1KLF2	Custom TaqMan Assays*
KLF2 endo	Custom TaqMan Assays*
NANOG endo	Custom TaqMan Assays*

Endo probes detect 3′-UTR of endogenous NANOG and KLF2 transcripts. Transgene specific probes cover the junction between vector and cDNA.

UPL			
**Gene**	**Primer**	**Sequence**	**UPL probe**
STELLA	U_STELLA R	tggtagcaatttgaggctctg	#80
	U_STELLA L	atcggcgtcttgacacaac	
ISL1	U_ISL1 L	aaggacaagaagcgaagcat	#66
	U_ILS_1 R	ttcctgtcatcccctggata	

Genomic PCR

DNA was isolated by QIAamp mini kit (QIAGEN 51304). The Copy Number Assay was performed following manufacture’s protocol (Life Technologies). 20ng of DNA were used per assay with four technical replicates.

Genomic PCR Primers	
CAG R	ATTACCATGGGTCGAGGTGA
CAG L	AGAAAAGAAACGAGCCGTCA
**Copy Number Assay (Taqman)**	
BsdR	Mr00733720_cn
Reference Assay, hRNase P	4403326
Reference Assay, hTERT	4403316

Immunostaining

Cells were fixed in 4% paraformaldehyde for 10 min, then blocked with 2% donkey serum/PBS + 0.1% BSA + 0.1% Triton (PBSBT) for 2 hr. Primary antibodies were diluted in PBSBT and incubated at 4°C overnight. Secondary antibodies were diluted 1:1000 and incubated at room temperature for 1 hr. Nuclei were counterstained with DAPI. Staining of methylated DNA was performed as previously described (Ficz et al., 2013). Cells were fixed by 4% PFA for 10 min. After permeabilisation by PBS+0.5% Triton for 1 hr, fixed cells were incubated in 2N HCl for 30 min, washed and then and then blocked in PBSBT for 2 hr. Cells were incubated in 1:250 5mC (Eurogentec BI-MECY) and 1:500 5hmC (Active Motif 39769)/PBSBT. Nuclei were stained with DAPI. Antibody details are provided below.

Antibody	Company	Cat NO	Dilution
NANOG	Abcam	ab21624	1:200
NANOG	eBioscience	14-5769-82	1:200
KLF4	Santa Cruz	sc-20691	1:400
TFCP2L1	R and D	AF5726	1:500
TFE3	Sigma	HPA023881-100UL	1:500
STELLA	Millipore	MAB4388	1:200
ECAD	Beckman Coulter	IM1763	1:100
CXCR4	BD Pharmingen	555974	1:100
SOX17	R and D	AF1924	1:200
FOXA2	Abnova	H00003170	1:200
TuJ1	R and D	MAB1195	1:200
NEUN	Millipore	MAB377	1:100
5-hmC	Active Motif	39769	1:500
5-mC	Eurogentec	BI-MECY-0100	1:250
H3K9me3	active motif	39765	1:500
ERK1/2	Cell Signaling	#9107	1:1000
pERK1/2	Cell Signaling	#4376	1:1000
alpha Tublin	Abcam	ab7291	1:5000

Flow Cytometry

Following dissociation with accutase or trypsin/EDTA, cells were blocked in donkey serum on ice for 20 min. Cells were stained on ice with E-cadherin antibody and CXCR4 antibody conjugated with PE in HBSS (Invitrogen) with 1% BSA for 20 min. After washing, APC Rat Anti-Mouse IgG1 secondary antibody (BD Pharmingen 550874) was applied. Flow cytometry analyses were performed using a Dako Cytomation CyAn ADP high-performance cytometer with Summit software.

Cell Metabolism Assays

Oxygen consumption was measured using an XF^{e}24 Analyzer (Seahorse Bioscience) according to the manufacturer's protocol. In brief, Seahorse plates were pre-treated by coating with laminin and 80,000 cells were seeded on each well the night before the experiment. Culture media were exchanged for XF Base Medium (Seahorse Bioscience) supplemented with 2mM pyruvate and 20mM glucose with an adjusted pH of 7.4 and cells incubated at 37°C in atmospheric CO_2 for one hour. Oligomycin (2 μM), FCCP (500 μM), antimycin (1 μM) and rotenone (1 μM) were injected during the assay (XF cell mito stress test kit, Seahorse Bioscience). Mitochondria were stained with MitoTracker Green FM (final concentration 50nM, Life Technologies) or tetramethylrhodamine, ethyl ester (TMRE, final concentration 20nM, Life Technologies) in the relevant medium for 10 min and analyzed by confocal microscopy.

Mass Spectrometry of Nucleosides

Genomic DNA was digested using DNA Degradase Plus (Zymo Research) according to the manufacturer's instructions and analyzed by liquid chromatography-tandem mass spectrometry on a Q-Exactive mass spectrometer (Thermo Scientific) fitted with a nanoelectrospray ion source. Mass spectral data for C, 5mC and 5hmC were acquired in selected reaction monitoring (SRM) mode, monitoring the transitions 228 → 112.0505 (C), 242 → 126.0662 (5mC) and 258 → 142.0611 (5hmC). Parent ions were isolated using a 1 mass unit window, fragmented with a normalised collision energy of 10%, and MS/MS spectra recorded with a resolution >45,000 for the fragment ions. Peak areas for the fragment ions were obtained from extracted ion chromatograms of the relevant scans and quantified by external calibration relative to standards obtained by digestion of nucleotide triphosphates.

BS-Seq Library Preparation and Analysis

Genomic DNA was prepared using AllPrep DNA/RNA mini kit (QIAGEN), fragmented by sonication (Covaris) and ligated to methylated adapters (Illumina) with the NEBnext library preparation kit (New England BioLabs). DNA was subsequently bisulfite-treated using the Sigma Imprint kit according to the manufacturer's instructions (one step protocol). Final library amplification (11 cycles) was performed with KAPA Uracil+ (Kapa Biosystems), after which the libraries were purified using 1x Ampure beads. Sequencing reads were filtered to remove low-quality calls and adapters were removed using v0.2.2 of Trim Galore (www.bioinformatics.babraham.ac.uk/projects/trim_galore) with default parameters. The remaining sequences were mapped to the human reference genome GRCh37 using Bismark v0.7.4 (Krueger and Andrews, 2011) with default parameters, and CpG methylation calls were extracted and analyzed using SeqMonk (www.bioinformatics.babraham.ac.uk/projects/seqmonk) and custom R scripts. Global methylation comparison was calculated by averaging 1kb window methylation levels of CpGs covered by at least 30 reads. To determine CGI methylation percentages probes were generated over CGIs and filtered for a minimum of 1 methylation count/CG and at least 5 CGs/CGI. Methylation values represent the mean over each CGI, filtered by chromosome.

RNA Processing

Total RNA was extracted with the TRIzol/chloroform method (Invitrogen), followed by resuspension in RNAsecure (Ambion), incubation with TURBO DNase (Ambion) at 37°C for 1 hr, further phenol/chloroform extraction and ethanol precipitation. RNA integrity was assessed with the RNA 6000 Nano assay on the 2100 Bioanalyzer (Agilent).

Transcriptome Sequencing

Ribosomal RNA was depleted from 5 μg of total RNA using Ribo-Zero capture probes (Epicenter). RNA samples were sheared by ultrasonication on a Covaris S2 for 90 s set at Duty Cycle 10, Cycles per Burst 200 and Intensity 5. Fragmented RNA was reverse-transcribed with a combination of random hexamer and oligo-dT primers (New England Biolabs) by SuperScript III (Invitrogen) at 50°C for 2 hr in the presence of 6 μg/ml actinomycin D (Sigma) to inhibit second-strand products. Second-strand cDNA was synthesized by DNA Polymerase I in the presence of RNase H with dUTPs substituted for dTTPs at 16°C for 2 hr. Sequential end repair and 3′-adenylation of cDNA products was carried out with T4 DNA polymerase and T4 polynucleotide kinase (20°C), and with exo$^-$ Klenow fragment (65°C) in the presence of dATPs (New England BioLabs). These were ligated to barcoded adapters (NEXTflex-96, Bioo Scientific) by T4 DNA ligase (New England BioLabs) at 20°C for 30 min. Second-strand DNA was digested with uracil DNA glycosylase (UDG) and Endonuclease VIII at 37°C for 30 min. PCR amplification of first-strand library constructs was carried out with KAPA HiFi DNA polymerase (Kapa Biosystems) for 13 cycles. Purification of reaction products at each step was performed with Ampure XP paramagnetic beads (Beckman Coulter). Library size distribution and molarity was assessed by the DNA 1000 assay on the 2100 Bioanalyzer (Agilent). Sequencing was performed on the Illumina HiSeq 2000 in 100bp paired-end format.

Microarray Processing

Total RNA was extracted and DNase treated as above, then processed for microarray analysis using the Ambion WT Expression Kit. Briefly, double-stranded cDNA was synthesized from 500 ng of RNA with random hexamers tagged with a T7 primer. Products were subjected to in vitro transcription by T7 RNA polymerase to generate antisense cRNA. Samples were reverse-transcribed by SuperScript III (Invitrogen) in the presence of dUTPs to yield single-stranded DNA. The template cRNA was then degraded by RNase H and cDNA products were fragmented by uracil DNA glycosylase (UDG) and apurinic/apyrimidinic endonuclease 1 (APE 1) (Ambion). Fragmented cDNA was then biotin-labeled by terminal deoxynucleotidyl transferase (TdT). Affymetrix Human Gene Array 1.0 ST arrays were hybridized for 16 hr at 45°C, washed, stained with streptavidin-phycoerythrin (SAPE) conjugate on a FS450 automated fluidics station (Affymetrix), and imaged on a GCS3000 7G scanner (Affymetrix).

RNAi

siRNAs (QIAGEN, below) were transfected at a final concentration of 40nM using Dharmafect 1 (Dharmacon T-2001-01), following the manufacturer's protocol. For a 24-well plate (2cm^2), we used 1 μl of transfection reagent in 50 μl of OptiMEM (Invitrogen), 1 μl of 20 μM siRNA solution in 50 μl of OptiMEM, and 4000 conventional PSC in 0.4ml of FGF/KSR medium or 2000 reset cells in 0.4ml of t2iL+ Gö. Medium was changed after overnight incubation.

si RNA List (QIAGEN)

Gene	No	siRNA	Cat No
GFP		GFP-22 siRNA	1022064
TFCP2L1	1	Hs_TFCP2L1_5	SI04206111
	2	Hs_TFCP2L1_3	SI00743253
	3	Hs_TFCP2L1_7	SI04312217
	4	Hs_TFCP2L1_6	SI04230625

(Continued on next page)

Continued

si RNA List (QIAGEN)			
Gene	No	siRNA	Cat No
REX1	1	Hs_ZFP42_7	SI04280241
	2	Hs_ZFP42_6	SI04221385
	3	Hs_ZFP42_9	SI04306974
	4	Hs_ZFP42_8	SI04304440
STELLA	1	Hs_DPPA3_1	SI00373233
	2	Hs_DPPA3_3	SI00373247
	3	Hs_DPPA3_8	SI04177642
	4	Hs_DPPA3_7	SI03204705
KLF4	1	Hs_KLF4_5	SI03176733
	2	Hs_KLF4_6	SI03649191
	3	Hs_KLF4_4	SI00463253
	4	Hs_KLF4_7	SI04144049

No 1 and 2 of were used for single siRNA knock down based on measurement of knock down efficiency by qRT-PCR.

shRNAs (Thermo Scientific, below) were introduced using the Neon Transfection System (Invitrogen), program 14 for conventional PSC and Neon program 20 for reset cells with 2 μg of shRNA vector. Two days after electroporation, cells were selected in puromycin. For rescue experiments, shRNA knockdown cells were transfected using Neon program 14 with 1.5 μg of piggyBac vector carrying a *Tfcp2l1, KLF4* or *ESRRB* expression cassette plus 1.5 μg of pBase and selected in hygromycin.

TRC human shRNA Thermo scientific		
Gene	clone ID	
TFCP2L1	TRCN0000017760	TFCP2L1_3
TFCP2L1	TRCN0000017762	TFCP2L1_5
KLF4	TRCN0000005313	KLF4_11*
KLF4	TRCN0000010934	KLF4_15
PKCl/i	TRCN0000006037	PKCi1
PKCl/i	TRCN0000006040	PKCi4
PKC zeta	TRCN0000010114	PKCz7
Empty Vector Control	RHS4080	

*3′ UTR

SUPPLEMENTAL REFERENCES

Aflatoonian, B., Ruban, L., Shamsuddin, S., Baker, D., Andrews, P., and Moore, H. (2010). Generation of Sheffield (Shef) human embryonic stem cell lines using a microdrop culture system. In Vitro Cell. Dev. Biol. Anim. *46*, 236–241.

Krueger, F., and Andrews, S.R. (2011). Bismark: a flexible aligner and methylation caller for Bisulfite-Seq applications. Bioinformatics *27*, 1571–1572.

Marson, A., Levine, S.S., Cole, M.F., Frampton, G.M., Brambrink, T., Johnstone, S., Guenther, M.G., Johnston, W.K., Wernig, M., Newman, J., et al. (2008). Connecting microRNA genes to the core transcriptional regulatory circuitry of embryonic stem cells. Cell *134*, 521–533.

Moretti, A., Bellin, M., Jung, C.B., Thies, T.M., Takashima, Y., Bernshausen, A., Schiemann, M., Fischer, S., Moosmang, S., Smith, A.G., et al. (2010). Mouse and human induced pluripotent stem cells as a source for multipotent Isl1+ cardiovascular progenitors. FASEB J. *24*, 700–711.

Thomson, J.A., Itskovitz-Eldor, J., Shapiro, S.S., Waknitz, M.A., Swiergiel, J.J., Marshall, V.S., and Jones, J.M. (1998). Embryonic stem cell lines derived from human blastocysts. Science *282*, 1145–1147.

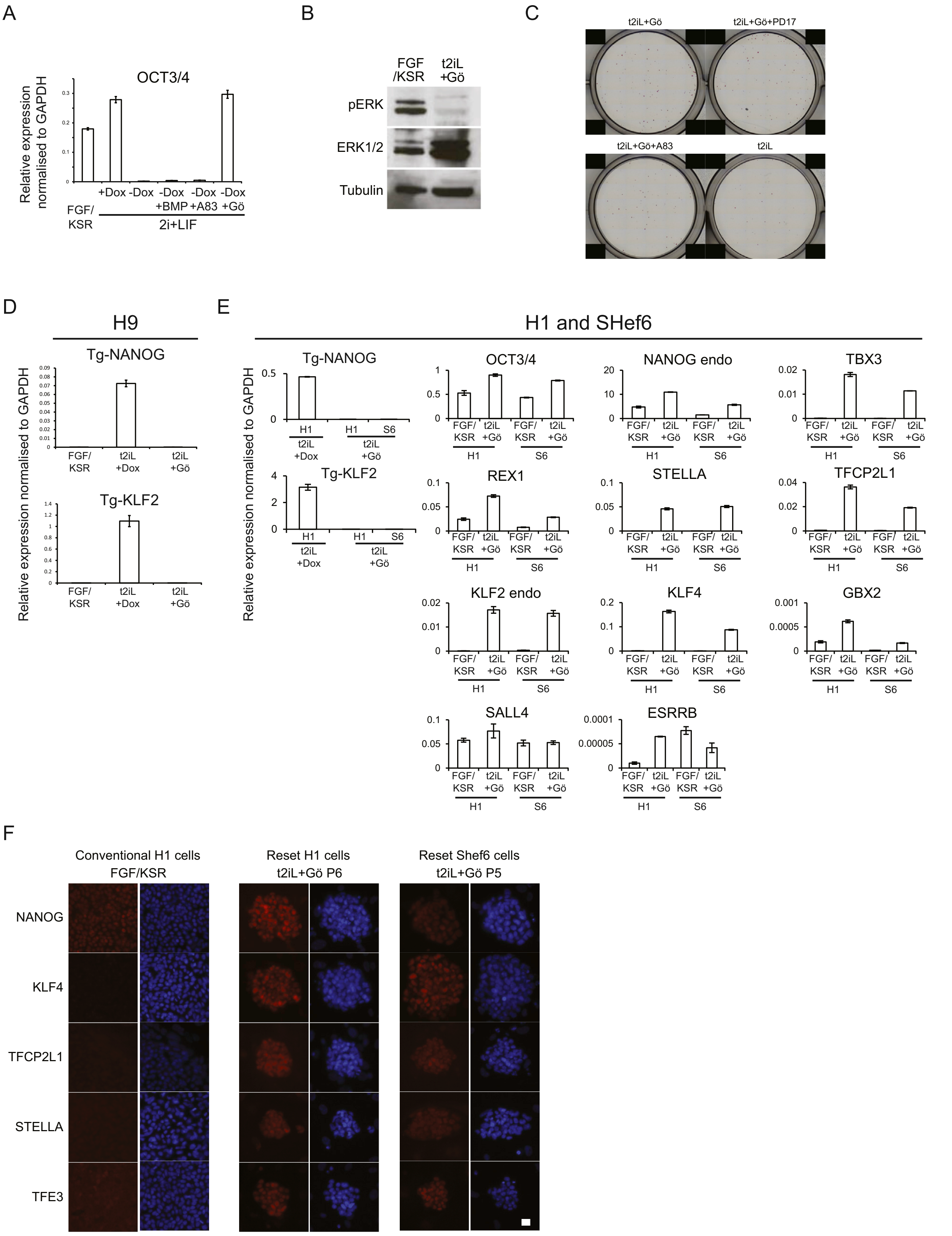

(legend on next page)

Figure S1. Resetting Human PSC, Related to Figure 1

(A) OCT4 expression after withdrawal of DOX. OCT4 mRNA expression was measured by qRT-PCR.

(B) Immunoblotting for ERK1/2 and pERK1/2. Protein was extracted from H9 cells cultured in FGF/KSR or reset in t2iL+Gö from a single well of a 6-well plate (1×10^6 cells). One fifth of the sample was fractionated by SDS electrophoresis, electroblotted and probed with indicated antibodies.

(C) Image of colony forming assay quantified in Figure 1E. Reset H9 cells maintained in t2iL+Gö were seeded without ROCKi at 2000 cells/well in 12-well plates in t2iL+Gö. FGF receptor inhibitor PD173074 (0.5 μM) or TGF-β/activin receptor inhibitor A83-01 (0.25 μM) were added as indicated. Colonies were stained for alkaline phosphatase after 7 days.

(D) Transgene-specific qRT-PCR assay. H9 cells harboring DOX-inducible *NANOG* and *KLF2* transgenes were assayed in the indicated culture conditions.

(E) Expression of ground-state transcription factor transcripts. qRT-PCR assay on reset H1 and Shef6 cells.

(F) Immunostaining for ground-state pluripotency markers. Conventional and reset H1 and Shef6 cells were stained with antibodies against the indicated markers. Note nuclear localization of TFE3 in reset cells.

Error bars indicate SD.

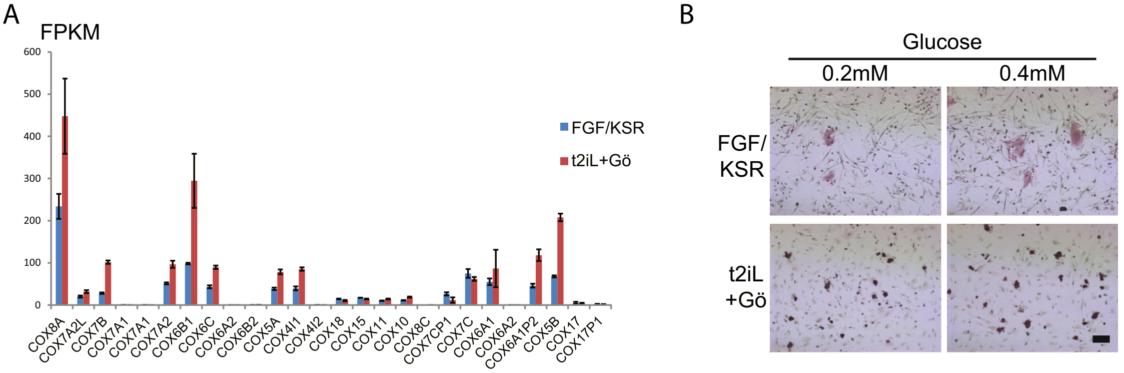

Figure S2. Mitochondrial Activity, Related to Figure 3

(A) COX gene expression determined from RNA-seq analysis. Data extracted from sample analysis in Figure 5

(B) Proliferation in low glucose. After single-cell dissociation, $3x10^4$ cells were seeded on 12-well plates and cultured for 7 days in the indicated concentrations of glucose. ROCKi was added for seeding conventional PSC. Conventional PSC failed to generate colonies. Reset cells are tolerant against low glucose produce multiple colonies. Scale bars: 200 μM.

Error bars indicate SD.

A

BS-seq Sample	Sequencing reads	Mapping Efficiency (%)	Fold coverage (Genome length GRCh37.p13)
Conventional hPSC H9 1	163350819	84.9	4.17
Conventional hPSC H9 2	156765441	85.1	4.00
Conventional hPSC H9 3	153635899	84.9	3.92
Reset cells H9 1	154802363	67.1	3.12
Reset cells H9 2	155956363	66.0	3.09
Reset cells H9 3	137626880	65.8	2.72

B

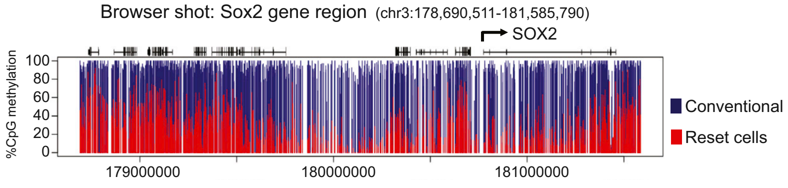

C

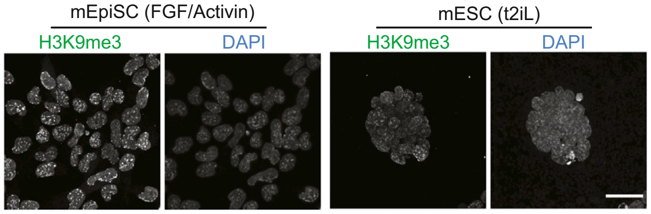

D

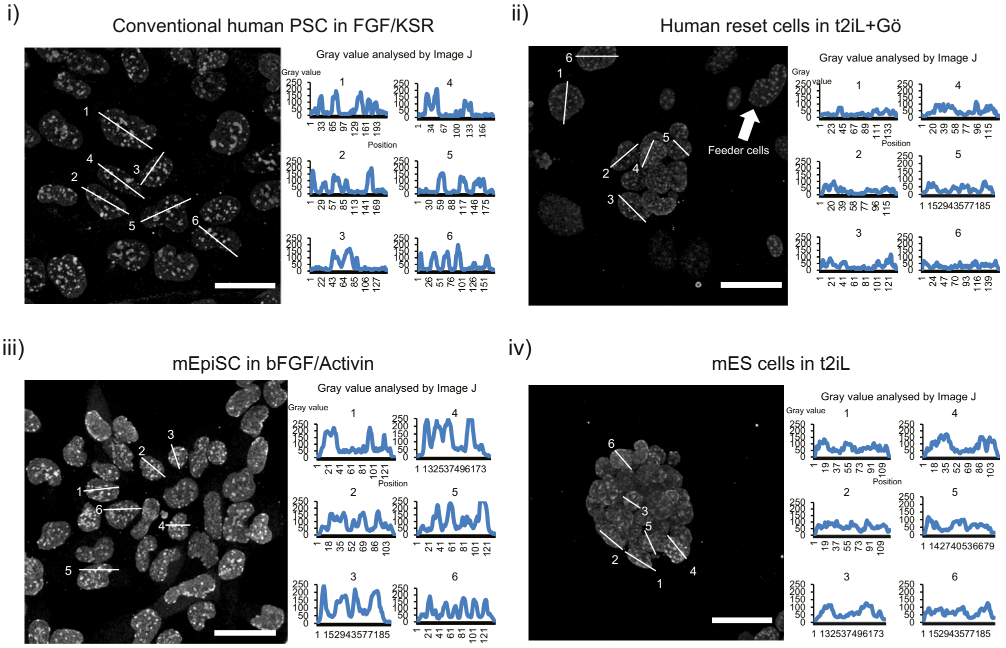

Figure S3. Epigenome Analysis, Related to Figure 4

(A) Sequencing coverage of whole-genome bisulfite sequencing libraries.

(B) BS-seq data for methylation at the *SOX2* locus in conventional versus reset H9 cultures.

(C) Immunofluorescence staining for H3K9me3 in mouse cells. Images of mouse post-implantation epiblast stem cells (EpiSC) in FGF/activin and mouse ESC in t2iL. H3K9me3 appears in green and DAPI staining in blue.

(D) Intensity and distribution of H3K9me3. Six cells were selected at random and intensity and distribution of staining were analyzed by Image J. i). Conventional human PSC in FGF/KSR. ii). Reset cells in t2iL+Gö. iii). Mouse EpiSC in bFGF/Activin. iv). Mouse ESC in t2iL.

Scale bars, 20 μM.

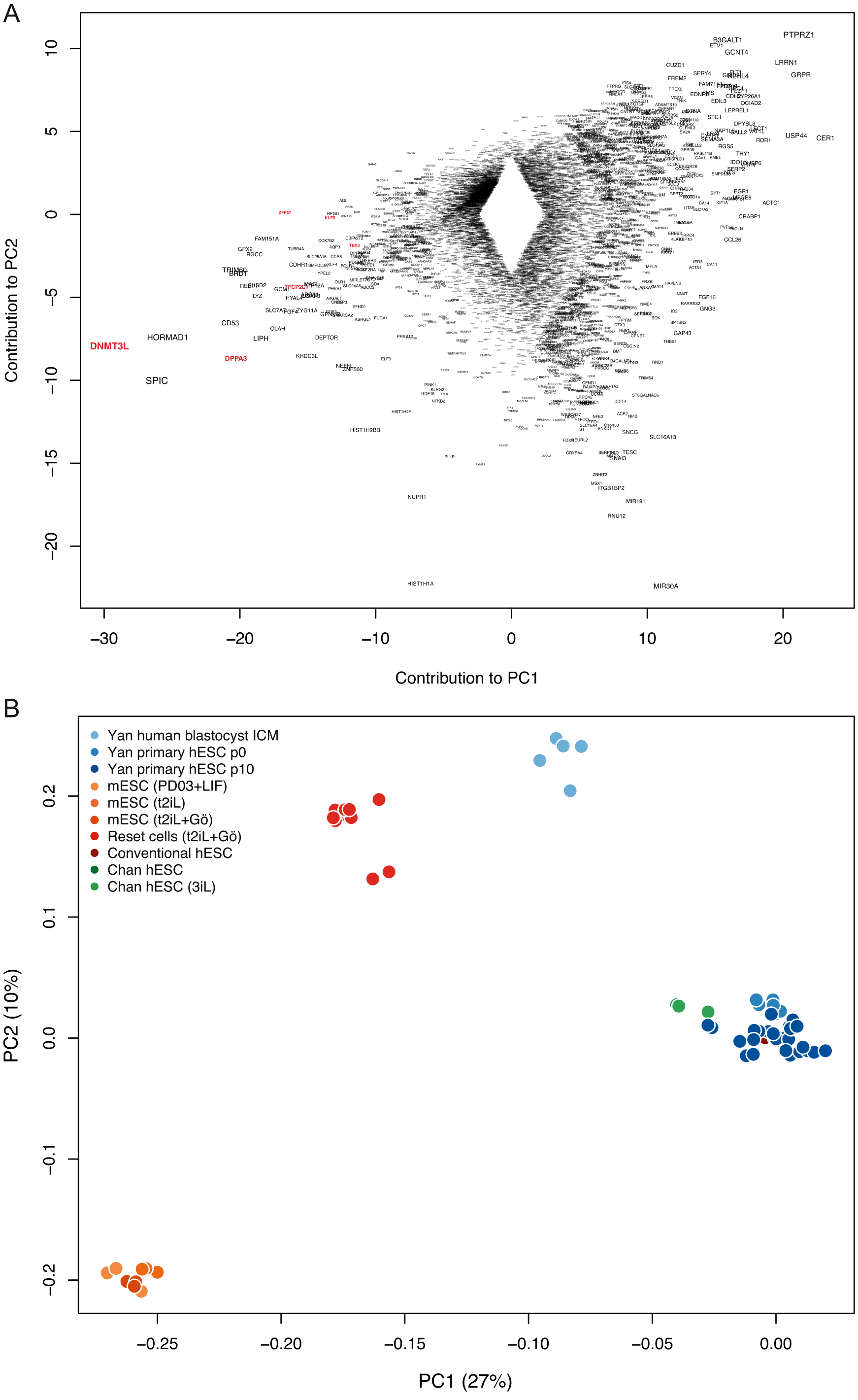

Figure S4. Comparative Expression Analysis, Related to Figure 5

(A) Genes contributing to principal components distinguishing reset cells from conventional human PSC. Gene symbols were extracted from the PCA and the labels scaled relative to the magnitude of variance. Pluripotency regulators are present in the leftmost area defining reset cells, whereas numerous lineage-specific genes can be found to the right expressed in conventional human PSC cultures.

(B) Platform-specific principal component analysis of RNA-seq data. Data are compared from reset cells, mouse ESC cultured in three different conditions, conventional human PSC and 3iL samples from Chan et al. Clustering of cell types when applied to a single technology recapitulates the integrated analysis in Figure 5A.

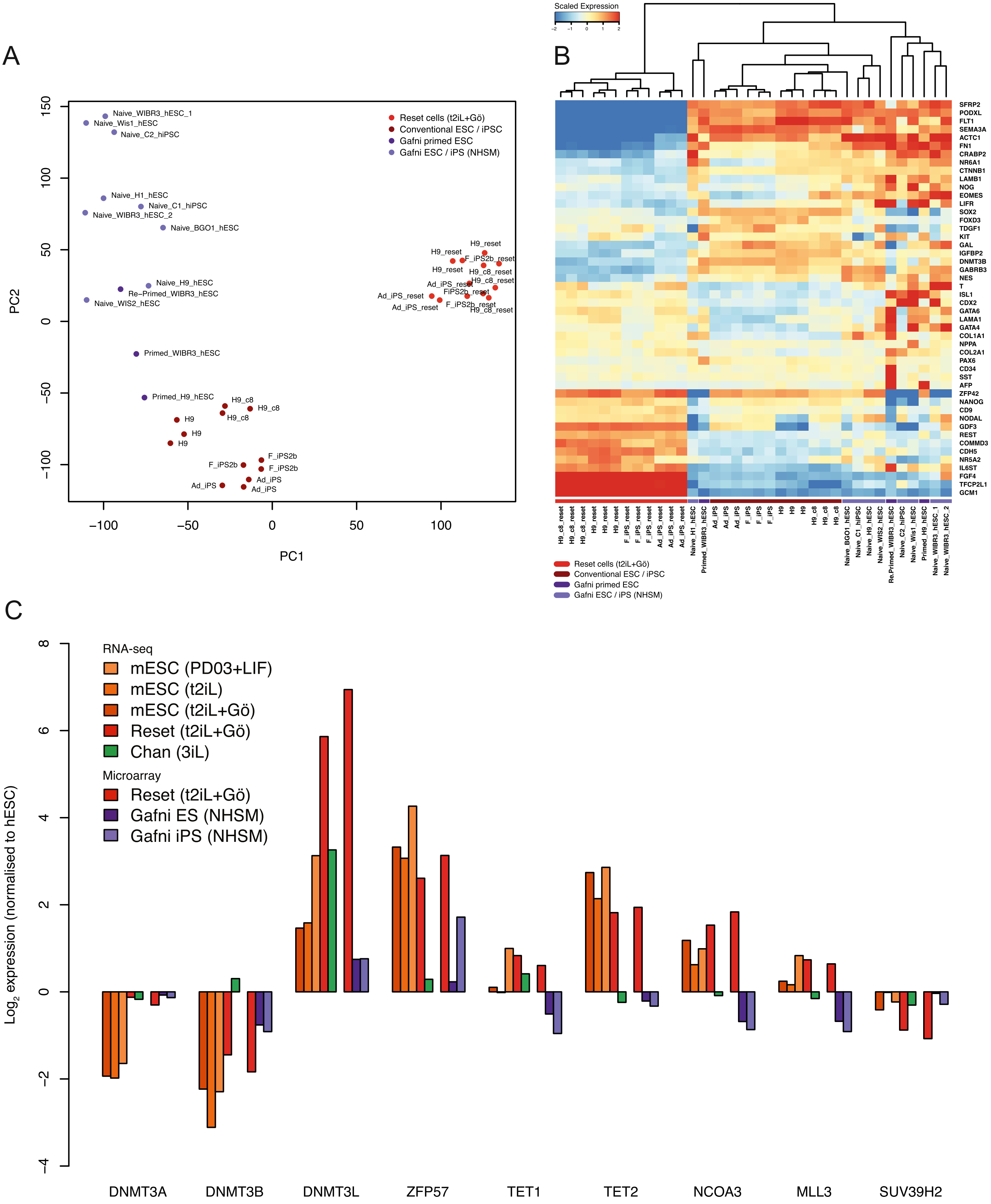

(legend on next page)

Figure S5. Marker Genes Distinguish Reset Cells from Conventional Human PSC and Alternative Protocols, Related to Figure 5

(A) Platform-specific principal component analysis of microarray data from this study and those reported in Gafni et al. Samples were hybridized to the same array platform to allow for direct comparison. Reset cells (light red) occupy a tight cluster to the right and conventional PSC (dark red) toward the bottom. Cells described as naive in Gafni et al. (violet) exhibit wide variation and appear unrelated to ground-state cells.

(B) Heatmap comparing the expression of 48 pluripotency and lineage marker genes selected by the International Stem Cell Consortium (Adewumi et al., 2007) between reset cells, conventional PSC cultures and those reported in Gafni et al., based on Affymetrix Human Gene 1.0 ST data. Reset cells form a distinct, relatively uniform population with robust expression of pluripotency genes and repression of lineage markers. In contrast, reportedly naive cells from Gafni et al. display many of the same traits as conventional PSC with mixed expression of lineage markers and significant reduction of key pluripotency regulators. Only genes for which a difference in expression was observed are displayed (i.e., scaled expression > 1 or < −1 in at least one sample).

(C) Panel of chromatin modification genes associated with DNA methylation and demethylation, histone methylation and acetylation. Expression trends in ground-state ESC are recapitulated in reset cells, whereas weaker or divergent transcription is evident in PSC cultured in alternative conditions. Expression levels are scaled relative to conventional PSC samples from each study. Data from different platforms are separated by spaces between bars.

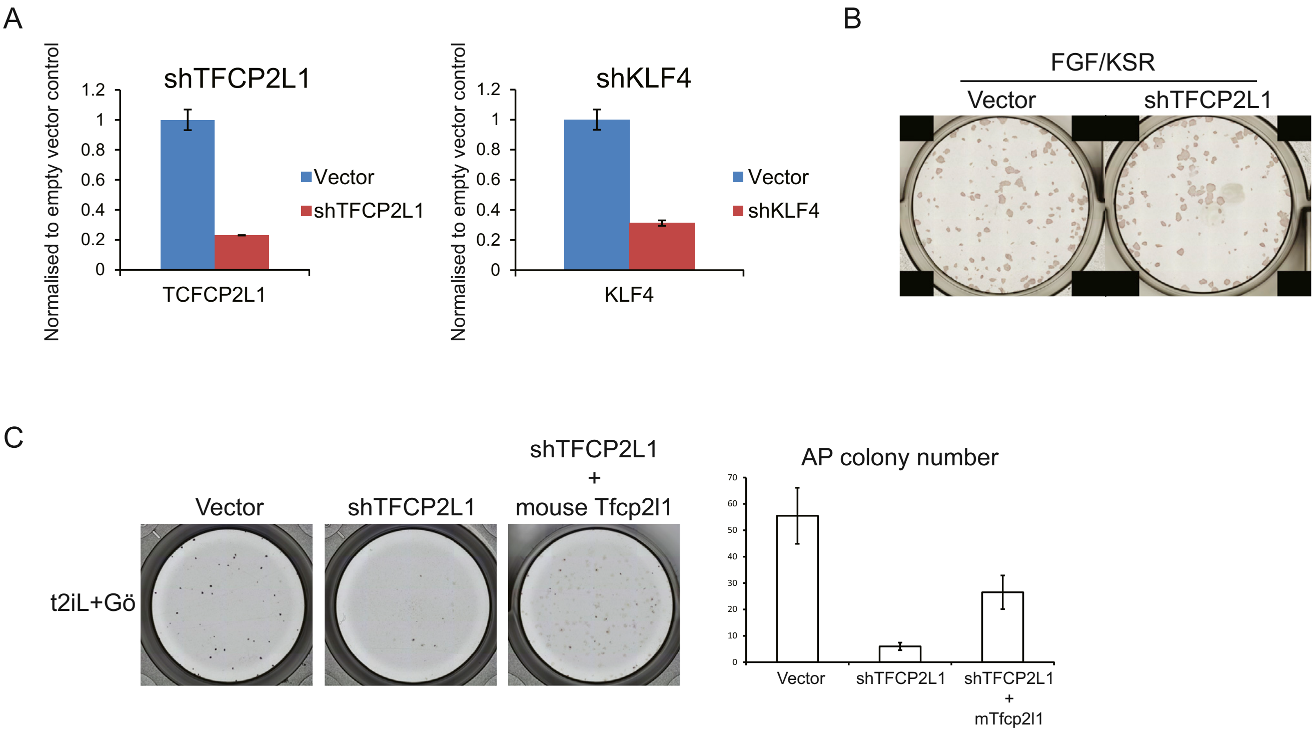

Figure S6. Functional Interrogation of Transcription Factor Circuitry, Related to Figure 6

(A) shRNA knockdown of *TFCP2L1* and *KLF4*. Knockdown cells and cells transfected with empty vector were maintained by expression of *NANOG* and *KLF2* transgenes in t2iL+DOX. Knockdown was evaluated by qRT-PCR.

(B) Colony formation after shTFCP2L1 knockdown (KD) in FGF/KSR. Parental H9 cells were stably transfected with empty vector or shTFCP2L1 construct and selected in puromycin. 4000 cells were plated in FGF/KSR with ROCKi in 12-well plates.

(C) Rescue of *TFCP2L1* knockdown with mouse *Tfcp2l1*. Colony formation by shTFCP2L1 knockdown cells transfected with mouse *Tfcp2l1* expression vector. Knockdown cells maintained by DOX induction of *NANOG* and *KLF2* were transfected with a piggyBac m*Tfcp2l1* expression vector and transfectant pools established by selection in hygromycin. Cells were then plated at 2000 cells/well in 24-well plates in t2iL+Gö without DOX and stained for alkaline phosphatase after 7 days.

Error bars indicate SD.

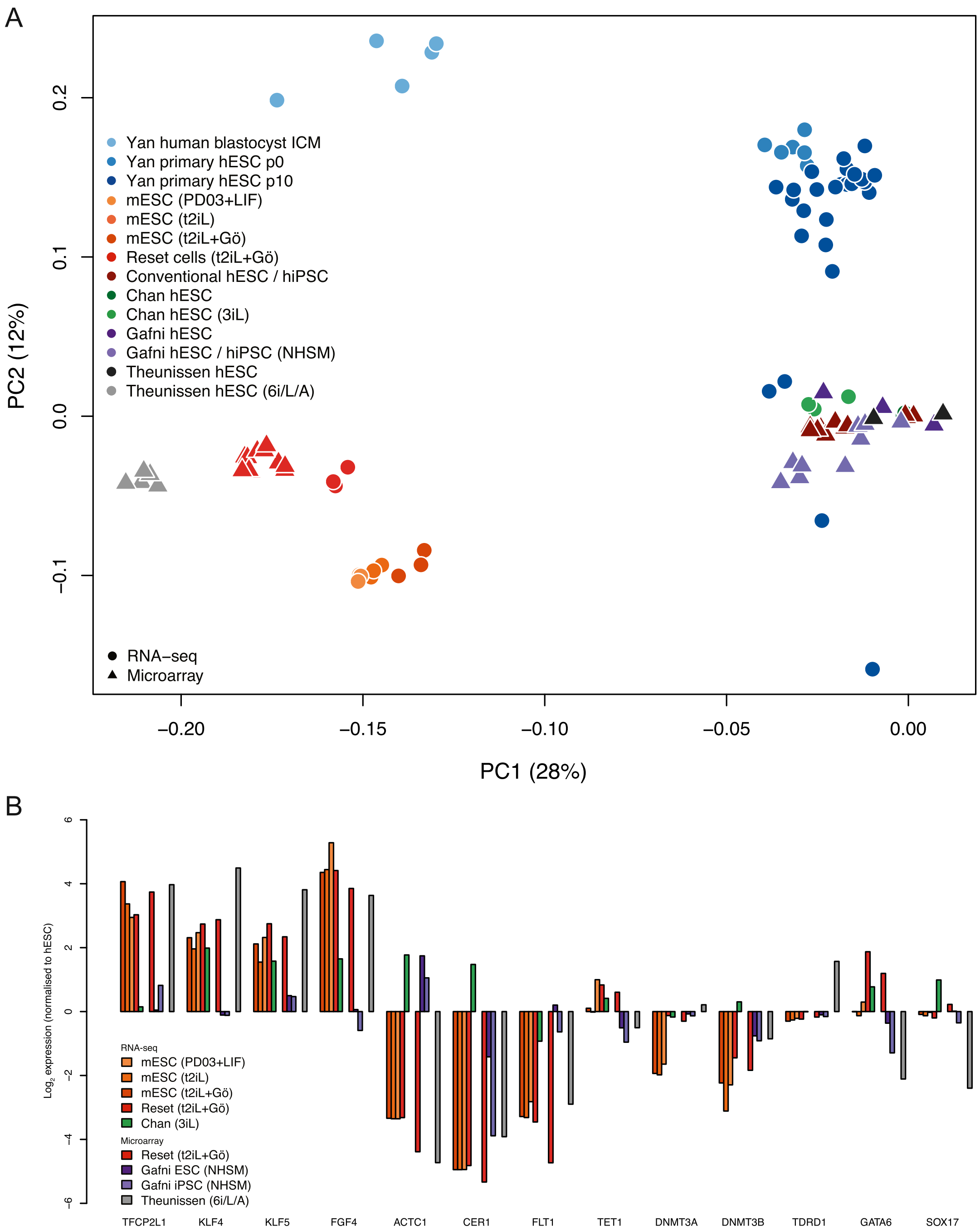

Figure S7. Transcriptome Meta-Analysis, Related to Discussion

(A) Principal component analysis of human and mouse PSC. Expression data were analyzed from conventional and 6i/L/A cultured human PSC (Theunissen et al.), H9 conventional and reset cells (this study), ground-state ESC cultured in three different conditions (this study), conventional and NHSM cultured human PSC (Gafni et al.), conventional and 3iL cultured human PSC (Chan et al.) and single cells from human blastocyst ICMs and primary explant cultures (Yan et al.). 6i/L/A samples share global similarity with reset cells but appear more divergent from mouse ESC.

(B) Comparison of individual human and mouse genes expressed in alternate culture conditions. Data are normalized to expression levels from conventional human PSC profiled on each platform. Notably, cells cultured in 6i/L/A largely recapitulate expression trends in reset cells and ground-state ESC, but reduced expression of TET1 and SOX17 and upregulation of DNA methyltransferase DNMT3A and germ cell marker TDRD1 indicate noteworthy differences, potentially reflecting developmental distinctions in pluripotent stage. Expression levels are scaled relative to conventional PSC samples from each study. Data from different platforms are separated by spaces between bars.

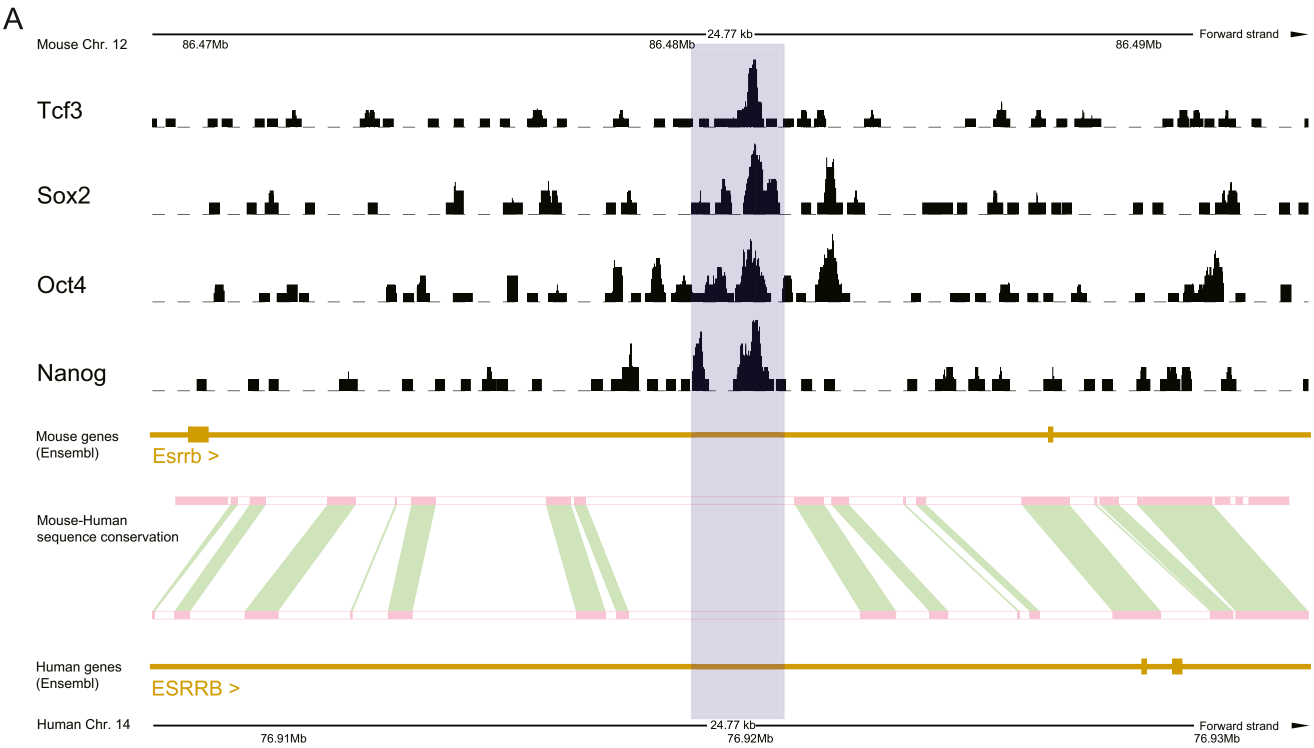

Figure S8. Incomplete Conservation between Mouse and Human *Esrrb* Loci, Related to Discussion

Esrrb locus of the mouse genome showing Nanog, Oct4, Sox2, and Tcf3 binding sites determined by ChIP-seq with sequences homologous to the equivalent human locus indicated. Signal tracks for ChIP-seq data from Marson et al. (Marson et al., 2008), were obtained from the the ES cell ChIP-seq compendium (Martello et al., 2012)

(A) Gray rectangle delineates the binding site of Tcf3, Sox2, Oct4 and Nanog in mouse where there is no conserved sequence between mouse and human. Conserved sequence is shown in red and green.

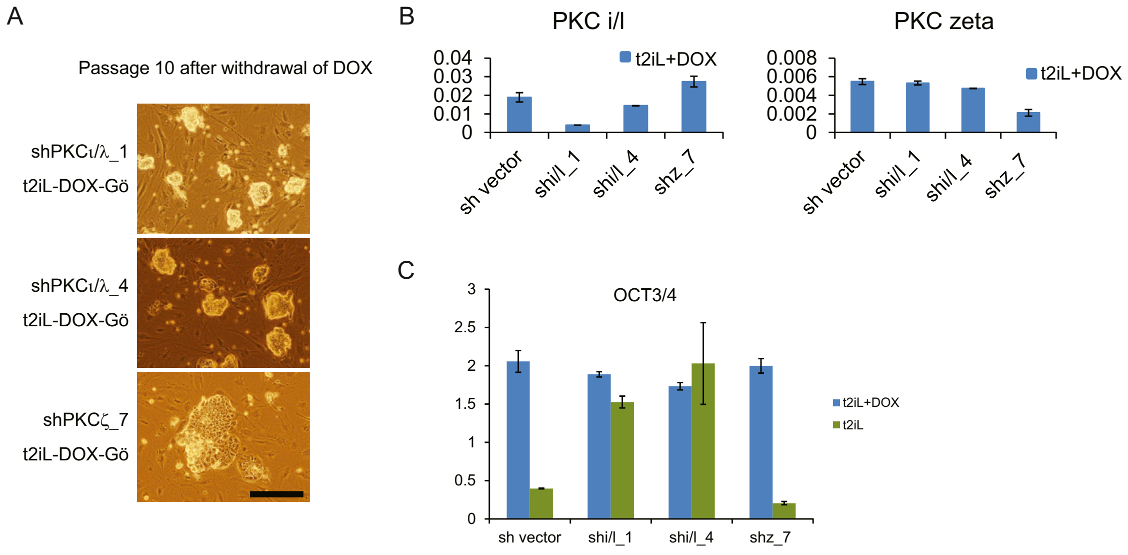

Figure S9. Knockdown of aPKC iota/lambda and zeta, Related to Discussion

Two different shRNA vectors, shPKC iota_1 and shPKC iota_4, were used for knockdown of PKC iota/lambda. shPKCzeta_8 was used for knockdown of PKC zeta. Scale bars: 100 μM.

(A) Brightfield images of shPKC iota KD and shPKC zeta KD cells cultured in t2iL. shPKC iota KD cell retain undifferentiated morphology whereas shPKC zeta cells progressively differentiate.

(B) Knockdown efficiency of each shRNA.

(C) OCT4 expression at passage 10 (KD lines) or 3 (control).

Error bars indicate SD.
